# Amyloid β Dodecamer Disrupts the Neuronal Membrane More Strongly than the Mature Fibril: Understanding the Role of Oligomers in Neurotoxicity

**DOI:** 10.1021/acs.jpcb.2c01769

**Published:** 2022-05-17

**Authors:** Hoang Linh Nguyen, Huynh Quang Linh, Pawel Krupa, Giovanni La Penna, Mai Suan Li

**Affiliations:** † Institute for Computational Science and Technology, SBI Building, Quang Trung Software City, Tan Chanh Hiep Ward, District 12, Ho Chi Minh City 729110, Vietnam; ‡ Ho Chi Minh City University of Technology (HCMUT), Ho Chi Minh City 740500, Vietnam; § Vietnam National University, Ho Chi Minh City 71300, Vietnam; ∥ Institute of Physics, Polish Academy of Sciences, Al. Lotnikow 32/46, Warsaw 02-668, Poland; ⊥ National Research Council of Italy (CNR), Institute for Chemistry of Organometallic Compounds (ICCOM), Florence 50019, Italy; # National Institute for Nuclear Physics (INFN), Section of Roma-Tor Vergata, Rome 00815, Italy

## Abstract

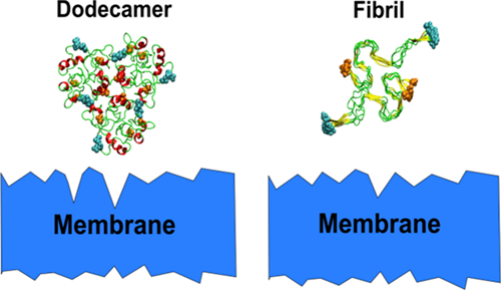

The amyloid cascade hypothesis states that senile plaques, composed of amyloid β (Aβ) fibrils, play a key role in Alzheimer’s disease (AD). However, recent experiments have shown that Aβ oligomers are more toxic to neurons than highly ordered fibrils. The molecular mechanism underlying this observation remains largely unknown. One of the possible scenarios for neurotoxicity is that Aβ peptides create pores in the lipid membrane that allow Ca^2+^ ions to enter cells, resulting in a signal of cell apoptosis. Hence, one might think that oligomers are more toxic due to their higher ability to create ion channels than fibrils. In this work, we study the effect of Aβ42 dodecamer and fibrils on a neuronal membrane, which is similar to that observed in AD patients, using all-atom molecular dynamics simulations. Due to short simulation times, we cannot observe the formation of pores, but useful insight on the early events of this process has been obtained. Namely, we showed that dodecamer distorts the lipid membrane to a greater extent than fibrils, which may indicate that ion channels can be more easily formed in the presence of oligomers. Based on this result, we anticipate that oligomers are more toxic than mature fibrils, as observed experimentally. Moreover, the Aβ–membrane interaction was found to be governed by the repulsive electrostatic interaction between Aβ and the ganglioside GM1 lipid. We calculated the bending and compressibility modulus of the membrane in the absence of Aβ and obtained good agreement with the experiment. We predict that the dodecamer will increase the compressibility modulus but has little effect on the bending modulus. Due to the weak interaction with the membrane, fibrils insignificantly change the membrane elastic properties.

## Introduction

Alzheimer’s disease (AD) is one of the most common forms of dementia.^1^ After decades of active research, the cause of this disease is still not clear, mainly due to the complexity of the human brain and the many factors that can influence its correct function. About 20 hypotheses have been developed,^2^ including amyloid β (Aβ) and tau protein modifications, Ca^2+^ imbalance, inflammation, cholinergic neuron damage, and oxidative stress. Among them, the amyloid cascade hypothesis,^3^ which posits that the senile plaques of Aβ peptides trigger AD, is one of the most promising hypotheses.^4,5^ The senile plaques are composed of highly ordered Aβ fibrils with a cross-β structure.^6^ However, many experimental studies have shown that the presence of senile plaques does not correlate with early neuronal loss,^4,7−9^ suggesting that fibrils are not the main toxic species. Instead, soluble agents formed during Aβ aggregation, called oligomers, can be considered the main cause of AD.^4,5,8−12^

The Aβ peptide, cleaved from the proteolytic amyloid precursor protein by β and γ-secretases,^13^ consists of 38–43 residues, but the most investigated alloforms are Aβ40 and Aβ42 containing 40 and 42 residues, respectively. The Aβ monomer does not have a stable conformation as Aβ belongs to the class of intrinsically disordered proteins/peptides. They can aggregate to form oligomers, protofibrils, and mature fibrils.^13^ Aβ42 is more prone to aggregation than the shorter Aβ40 alloform, although Aβ40 is more abundant than Aβ42. It is believed that toxic oligomers are off-pathway products of the aggregation process and consist of 2–50 monomers.^13,14^ In solution, oligomers are polymorphic and lack a stable structure, while mature fibrils are organized into cross-β structures.^15^

One of the most important questions is why Aβ oligomers are more toxic than mature fibrils. To at least partially answer this question, let us recall the molecular mechanism of AD-induced neurotoxicity, which is related to the interaction of Aβ with the neuronal membrane altering its permeability.^16,17^ According to this hypothesis, similar to pore-forming toxins,^18^ Aβ peptides form pores in the membrane that allow Ca^2+^ ions to pass through the membrane, resulting in neurotoxicity.^19,20^ In general, such channels can disrupt cellular homeostasis,^21,22^ induce the production of reactive oxygen species, and alter the signaling pathways^23^ and mitochondrial function.^24^

An alternative way to model pore formation is to place Aβ peptides on the membrane surface and monitor how they perforate the membrane using molecular dynamics (MD) simulations. However, the process of channel formation is slow, making this approach impractical with the existing computational facilities. A computationally more realistic approach is to insert a pre-formed channel, which consists of several β-strands or a barrel, into the membrane^25−27^ and study the stability of the complex. The disadvantage of this approach is that it does not completely solve the channel formation problem.

In this paper, we try to understand why oligomers are more toxic than fibrils by comparing their interactions with the membrane. For a case study, we have chosen the dodecamer because the experiment of Economou et al. showed that the Aβ42 hexamer and dodecamer are dominant and can serve as a seed for fibril formation.^28^ Moreover, Bernstein et al. found that dodecamer, which is the smallest long-living soluble oligomer for Aβ42, is a primary toxic species.^29^ Recent experiments have also shown that cell toxicity is associated with nanoparticles of about 4 nm in height.^30^ Such nanoparticles fit with annular assemblies of three tetramers or dodecamers. For comparison with the dodecamer, we used a model of mature fibrils which have 12 Aβ42 chains.

Different lipid bilayer models such as POPC, DOPC, and so forth have been used to study the Aβ–membrane interaction,^25,27,31,32^ but these homogeneous models can lead to artifacts because many lipid molecules are relevant for AD. Ganglioside GM1 can have an impact on Aβ fibrillogenesis and membrane disruption,^33,34^ while the cholesterol level affects the production and pore formation of Aβ in membranes.^35−37^ Therefore, we used a more realistic neuronal membrane model which is called the disease 1 (D1) model introduced by Drolle et al.^38^ This model is composed of DPPC, POPC, sphingomyelin (SM), cholesterol, and ganglioside GM1,^39^ which are found in the outer leaflet of neuronal cell membranes.^40,41^ A different neuronal membrane model was used to study the dimerization of Aβ42 near the membrane using MD simulations.^42^ In this work, we also used all-atom MD simulations, as they were successful in characterizing Aβ monomers and low-weight oligomers in solution^43−45^ and on the membrane surface.^31,32^ We studied three systems, including pure membrane without Aβ, membrane–dodecamer complex, and membrane–fibril complex.

In the absence of Aβ, we were able to reproduce the experimental results on the bending and area compressibility modulus, as well as on the dependence of the bilayer thickness on cholesterol in a heterogeneous bilayer membrane. We showed that the elastic properties of the membrane change under the action of Aβ, especially in the case of the dodecamer. Due to the short timescales of simulations, we could not observe the formation of pores, but different effects of the oligomers and mature fibrils on the early events of this process were obtained. We showed that the oligomer interacts with the membrane more strongly than the fibril. Consequently, in the presence of the dodecamer, the membrane is damaged to a greater extent than in the presence of fibrils, which may partially explain why oligomers are more neurotoxic. The electrostatic interaction energy between Aβ and the membrane was shown to play a key role in the influence of Aβ on membrane properties such as lipid distribution and the depth of cracks on the membrane surface. We have found that both oligomers and fibrils increase the area compressibility modulus of the membrane but leave the bending modulus almost unchanged.

## Materials and Methods

### Initial Structures of Aβ

**Figure 1:**
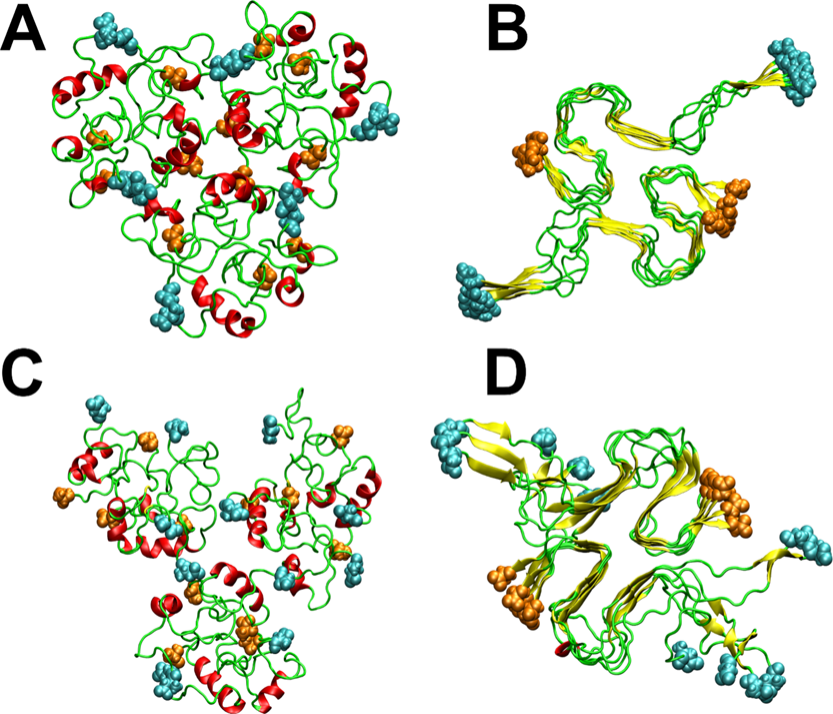
(A) Initial structure of the Aβ42 dodecamer obtained from the three tetramers using the docking method. (B) Mature fibril structure obtained by duplicating the PDB 2NAO structure, which consists of six chains. (C) Structure of a dodecamer obtained from the 500 ns MD run in solution. (D) As in C but for a mature fibril. The N-terminal and C-terminal atoms are shown with cyan and orange balls, respectively.

The initial structure of the Aβ42 dodecamer (Figure 1) was generated from the three Aβ42 tetramers obtained in our previous work,^44^ using the docking procedure implemented in the web server Galaxy.^46,47^ GalaxyGemini shows good performance in oligomeric structure prediction compared to other predictors.^47^ The input structure is searched to check if it is oligomeric or not. If an oligomeric state exists, its structure is predicted by superimposing the input monomer onto the oligomer structure template. The energy of the oligomer structure is then minimized to eliminate steric clashes at the interface of monomers.^47^ Since we are studying the behavior of the 12-mer near the membrane surface, our choice differs from the pore-forming aggregate developed by different groups^48,49^ for the case where Aβ are located inside the membrane.

To obtain a Aβ42 fibril structure with 12 chains, we duplicated the six-chain 2NAO PDB structure,^15^ displacing this structure along the fibril axis by 18.4 Å, which is the thickness of chains in the 2NAO structure (Figure 1).

### Model of a Neuronal Membrane

**Figure 2:**
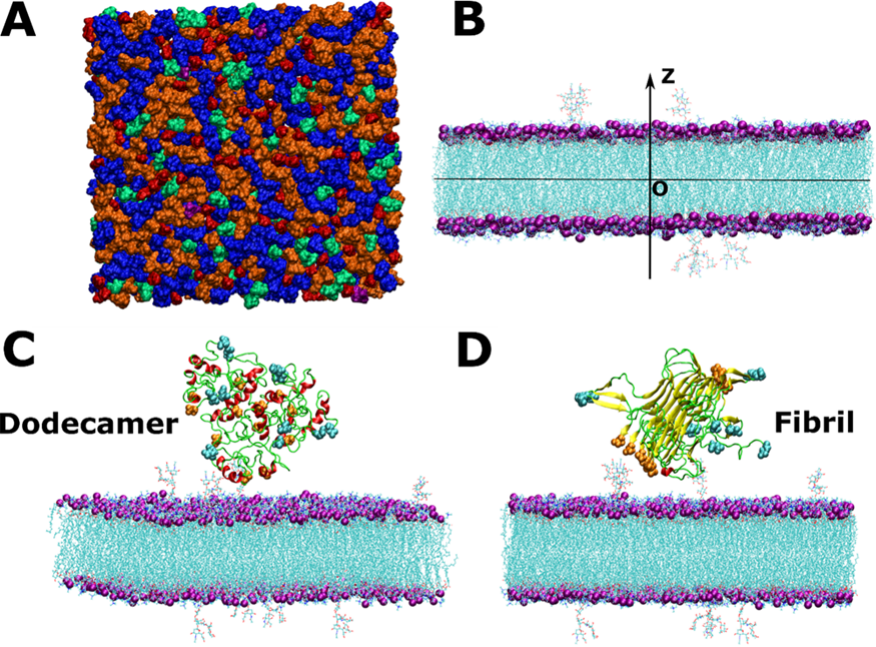
(A) Lipid molecules in the membrane (view in direction perpendicular to the membrane surface), DPPC—blue, POPC—orange, CHL1—red, PSM—green, and GM1—purple. (B) The *z*-axis is perpendicular to the surface of the membrane and *z* = 0 at the center of the membrane. (C) Typical initial conformation of the membrane–dodecamer complex, water, and ion molecules are removed for clarity. The P atoms of lipids are shown as purple balls, and the N-terminal and C-terminal atoms of the Aβ peptide are show as cyan and orange balls, respectively. (D) As in C but for the membrane–fibril complex. The Aβ structure shown in (C,D) was obtained from the 500 ns simulation in solution.

To mimic the cell membrane seen in an Alzheimer’s patient, we created a multi-lipid bilayer that consists of five types of lipids,^38^ DPPC–POPC–PSM–CHL1–GM1, and the molar ratios of these lipids are 36.5:35.2:9.7:17.8:0.8, respectively (Figure 2). DPPC and POPC belong to the PC lipid group, CHL1 belongs to the cholesterol group, PSM belongs to the SM lipid group, and GM1 belongs to the glycolipid group.

The GM1 concentration of 0.8% in our model is lower than 3–4% reported in previous works (see Fatafta et al.^42^ and references therein). The ganglioside and cholesterol (CHL1) concentrations in healthy and Alzheimer’s brains are determined in μmol/g in Svennerholm and Gottfries.^50^ The CHL1 concentration is about 13.1 times of the concentration of gangliosides in the brain with Alzheimer’s type I disease. Furthermore, the average CHL1 concentration in neuronal cells is about 17.8%.^51^ Based on these figures, the estimated concentration of gangliosides in the brain with Alzheimer’s type I disease is about 1.4%. However, in this brain, there are many types of gangliosides, such as GM1, GM2, GD3, GD1a, GD1b, GT1b, and GQ1b1, which means that the GM1 concentration should be below 1.4%. Therefore, the GM1 concentration of 0.8% in the model we are using is reasonable, in particular, for an Alzheimer’s brain where GM1 is reduced as the disease progresses.^50^

The bilayer was built using the CHARMM GUI web server^52^ with 484 lipid molecules in one layer.

### MD Simulations

The GROMACS 2020.2 package^53^ was utilized to carry out MD simulations. The peptide and membrane were parameterized by the CHARMM36m and CHARMM36 force fields, respectively.^54,55^ For the MD simulation in solution, the original structures of the dodecamer and fibrils were solvated in a dodecahedron box with water, and the minimum distance between the protein and the box was 3 nm. Counterions were added to neutralize the system. The box size was 16.3 × 16.3 × 11 nm and 16.3 × 16.3 × 16.3 nm, and the number of atoms is 306,279 and 316,896 for the oligomer and fibril, respectively. The system was then energy-minimized and subsequently equilibrated in *NVT* and *NPT* ensembles for 1 and 5 ns, respectively. Conventional unrestricted MD simulations for 500 ns were performed, and the final structure was used to simulate the interaction of Aβ with the membrane.

The structures of the dodecamer and fibrils obtained in the previous 500 ns MD run in solution were randomly rotated to obtain 10 initial conformations and placed on a bilayer membrane with the minimum distance between the Aβ aggregate and the membrane of 15 Å (Figures 2 and S1 in the Supporting Information). The thus-obtained Aβ–lipid complexes were solvated in TIP3P^56^ water, and counterions were added to neutralize them. A NaCl concentration of 150 mM was used to mimic physiological conditions. The dodecahedron box size was 16.5 × 16.5 × 17 nm and 16.5 × 16.5 × 17 nm, and the number of atoms was 441,534 and 440,253 for the oligomer and fibril, respectively. The energy was minimized by the steepest descent algorithm. The system was then equilibrated at 323 K and 1 atm by performing 2 and 5 ns MD simulations in *NVT* and *NPT* ensembles, respectively. Note that the temperature was chosen to be higher than the phase-transition temperature of PSM (*T*_M_ ≈ 314 K),^57^ cholesterol (304.8 K),^58^ GM1 (292.5 K),^59^ POPC (270 K),^60^ and DPPC (314 K).^61^ The temperature and pressure were preserved using v-rescale^62^ and Parrinello–Rahman^63^ algorithms. The particle mesh Ewald algorithm was used to calculate the electrostatic interaction energy with a cutoff of 1.2 nm.^64^ To calculate van der Waals (vdW) interactions, a double cutoff of 1.0 and 1.2 nm was chosen.

Replica exchange MD (REMD) is one of the best methods to obtain good sampling, but using it is beyond our computational capabilities since the membrane–Aβ complexes in explicit water consist of nearly half million atoms, and REMD would require to run dozens of trajectories (replicas) per system to ensure a proper exchange rate. Therefore, we carried out conventional MD simulations. After equilibration, we performed one MD trajectory for the membrane without Aβ and 10 trajectories for the membrane with the dodecamer and fibril, each starting with different conformations (Figure S1) and 800 ns per trajectory (8 μs per system).

### Elastic Modulus of the Membrane

Using the method proposed by Khelashvili et al.,^65^ we calculated the elastic modulus of the membrane from the splay modulus for lipid pair types. The bending modulus *K*_C_ was calculated from the equation
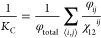
where χ_12_^*ij*^ is the splay modulus for the *ij*-th pair, φ_*ij*_ is the number of *ij* adjacent pairs, and φ_total_ is the total number of φ_*ij*_ for all pairs.^51^ To obtain the splay modulus χ, we performed a quadratic fit in the interval [10:30] degrees of the α angle of the function
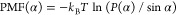
where α is the angle between a pair of lipid director vectors.

The director vector for lipids PC and SM is the vector from the midpoint between the P and C2 atoms to the center of mass of the last three carbon atoms on the two lipid chains. For cholesterol, the lipid director vector is a C3 to C17 vector.

### Area Compressibility Modulus

The area compressibility modulus was calculated from the area per lipid *A* using the equation
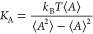
where ⟨...⟩ stands for averaging over snapshots sampled in equilibrium.

### Diffusion Coefficient for Lipid Molecules

The diffusion coefficient *D* of lipid molecules was calculated using the msd tool from the GROMACS package. It was extracted from the linear time dependence of the mean-square displacement (MSD)
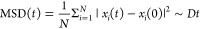
where *x*_*i*_ is the coordinate of atom *i* and *N* is the number of atoms.

### Acyl Chain Order Parameter

The acyl chain order parameter of lipid acyl tails (*S*_CD_) provides information of membrane order and the details of the conformations that the atoms within the lipid tails adopt. The order parameter shows the average orientation of inter-nuclear C–D vectors relative to the direction of the external magnetic field. This parameter is determined by the ^2^H NMR experiment. Furthermore, it correlates with membrane rigidity and area expansion modulus.^66^ Therefore, in order to investigate the effect of the Aβ42 dodecamer and fibril on the properties of lipid tail atoms and compare with the experimental data, we calculated the acyl chain order parameter. In this work, *S*_CD_ is defined as a measure of the orientation mobility of the C–H bond^66^
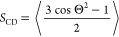
where θ is the time-dependent angle between the C–H bond vector and the *z*-axis (Figure S3). Angular brackets denote the time and ensemble average.

### Side Chain Contact

A side chain contact between two residues is formed when the distance between the centers of mass of their side chains is ≤6.5 Å.

## Results and Discussion

### Stability of the Initial Structure in Solution

We must first investigate the stability of the initial structure of the dodecamer and mature fibril in solution because for the dodecamer, it was obtained by combining the three tetramers obtained earlier,^44^ while for the fibril structure of 12 strands, it was built from the six-chain 2NAO PDB structure^15^ (Materials and Methods). The time dependence of root-mean-square deviation (rmsd), obtained from the 500 ns MD simulation for the dodecamer and fibril, is shown in Figure S3, which suggests that the system has reached equilibrium. Therefore, we will use the last structures (Figure 1C,D) as initial configurations for the 800 ns simulation with a neuronal membrane.

### Equilibration Time of Aβ–Membrane Complexes

Production runs of 800 ns were carried out starting with the initial configurations shown in Figure 2C,D. The time dependence of the Cα rmsd, radius of gyration (*R*_g_), number of contacts between chains, and total solvent-accessible surface area (SASA) indicate that systems became stable after 500 ns (Figure S4). Thus, the equilibration time of both Aβ–membrane complexes τ_eq_ ≈ 500 ns and the last interval [500–800] ns was chosen for data analysis.

### Dodecamer Is More Soluble than Fibril

The time dependence of the number of contacts between chains, gyration radius *R*_g_, and SASA of both complexes is shown in Figure S4B–D. Averaging over the time window [500, 800 ns] and 10 trajectories, we obtain 306 ± 15 contacts for the dodecamer, which is significantly less than 659 ± 5 of the fibril. In the presence of a membrane, dodecamer tends to increase its solubility observed by an increase in the SASA and *R*_g_ values, while the fibril becomes more compact and less soluble. At equilibrium, the mean SASA is 334.12 ± 3.16 and 268.14 ± 2.38 nm^2^ for the dodecamer and fibril, respectively, indicating that dodecamer is more exposed to water than fibril. This conclusion is also supported by the results obtained for *R*_g_, which is higher for dodecamer (2.70 ± 0.17 nm) than for fibril (2.35 ± 0.08 nm).

### Aβ42 Dodecamer Approaches the Membrane Closer than the Fibril

**Figure 3:**
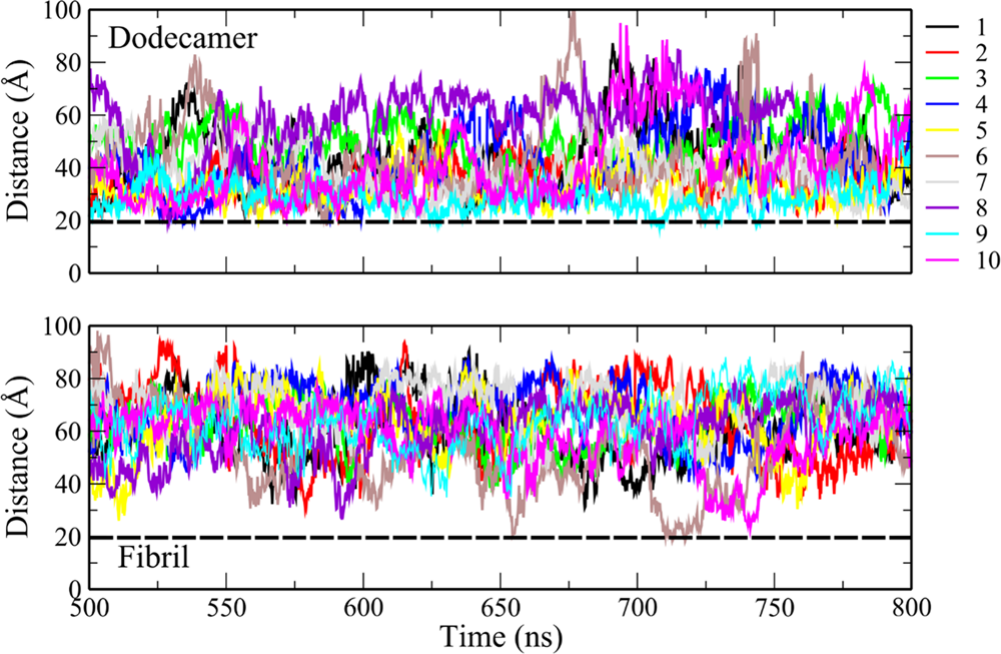
Minimum distance between Aβ and the center of the membrane along the *z*-axis as a function of time. The dashed line represents the membrane surface.

Figure 3 shows the time dependence of the minimum distance between Aβ and the center of the membrane along the *z*-axis. On trajectories 4, 5, 6, 7, 9, and 10 of the dodecamer–lipid system, many events occur when the oligomer crosses the surface of the lipid bilayer (minimum distance less than 2.06 nm, which is the half of the average membrane thickness). On the contrary, the fibril slightly crosses the membrane surface several times only in trajectory 6 (Figure 3). Thus, the Aβ42 dodecamer can approach the membrane surface closer than the mature fibril (Figure S5).

Assuming that Aβ forms a contact with the membrane if the minimum distance between their nearest atoms is less than 6.5 Å, we obtained the contact population for all MD trajectories (Table 1). We used the same cutoff as for the vdW interaction, which is small as the distance between two atoms exceeds 10 Å. For the dodecamer, the population exceeds 50% only for trajectory 5, implying the transient nature of the peptide–membrane contact. Figure S6 shows a typical snapshot in which part of the dodecamer can seep into the membrane but cannot penetrate deep into it during limited time of the simulation. This figure also demonstrates that the dodecamer, which enters slightly into the membrane, was not stable as it was poorly populated in our simulation (Figure 3).

**Table 1:** Frequency (%) of Snapshots with a Minimum Distance between the Aβ42 Dodecamer, Fibril, and Membrane Surface Is Equal to or Less than 10 Å

MD trajectory	dodecamer	fibril
1	16.15	0.0
2	3.27	0.5
3	33.53	0.0
4	10.40	0.0
5	56.18	1.0
6	12.66	8.9
7	25.80	0.0
8	3.96	1.3
9	21.52	0.0
10	10.13	5.5
average	19.4 ± 15.2	3.4 ± 3.2

In the case of fibrils, a maximum contact population of only 8.9% was observed in run 6. Therefore, the Aβ42 dodecamer interacts with the membrane and damages it to a greater extent (see below) than the mature fibril. This result is in qualitative agreement with experiments, showing that oligomers such as dodecamers are more toxic than mature fibrils.^67−69^ Although the dodecamer interacts with the neuronal membrane more strongly than the fibril, this interaction remains relatively weak, which is consistent with the experiment,^70^ reporting that Aβ oligomers interact weakly with lipid bilayers. Fibrils were found to interact with DOPC bilayers,^71^ and the detection of weak interactions described here might be due to membrane composition or short timescales of simulation.

### Electrostatic Interaction Energy between Aβ and the Membrane Is More Important than the vdW Interaction

**Figure 4:**
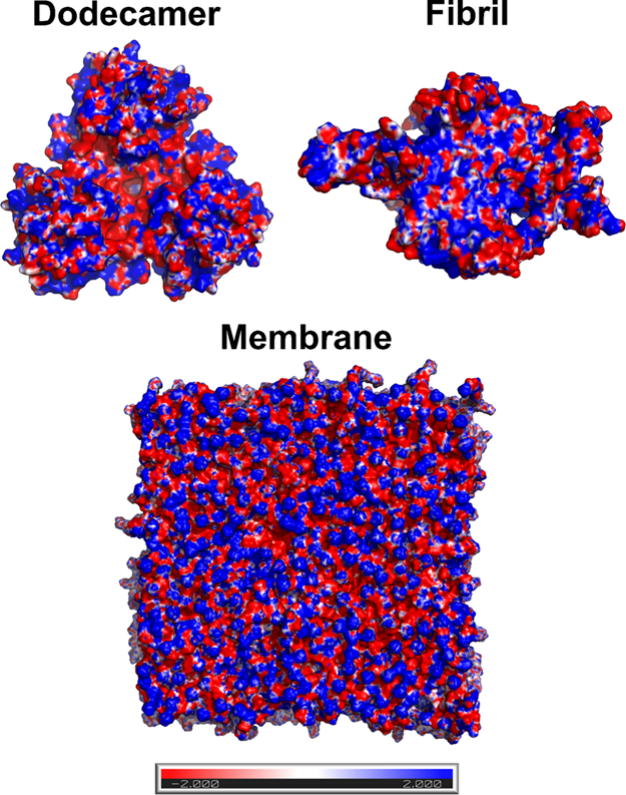
Electrostatic potential on the surface of dodecamer, fibril, and membrane. The color bar from red to blue indicates from negative to positive potential, respectively.

Using the configurations collected at equilibrium, we calculated the interaction energy between the peptides and the membrane (Table 2). The electrostatic interaction energy (in the range of 500–700 kcal) is much stronger than vdW (in the range of −20–0 kcal/mol) in both the dodecamer and the fibril. Strong repulsive electrostatic interaction energy prevents Aβ from entering the membrane without significant reorganization of the structure (Figure 4).

**Table 2:** Nonbonded Interaction Energy between Aβ and the Membrane (kcal/mol)^a^

trajectory	energy	Aβ42 dodecamer	Aβ42 fibril
1	electrostatic	634.5 ± 105.43	675.72 ± 77.45
	vdW	–1.70 ± 0.13	–0.16 ± 0.14
2	electrostatic	649.92 ± 134.91	633.40 ± 59.41
	vdW	–4.56 ± 0.70	–0.17 ± 0.12
3	electrostatic	675.32 ± 124.46	612.81 ± 55.53
	vdW	–0.62 ± 0.47	–0.09 ± 0.05
4	electrostatic	644.29 ± 67.84	586.19 ± 52.22
	vdW	–5.88 ± 0.53	–0.06 ± 0.04
5	electrostatic	587.43 ± 145.29	609.74 ± 82.76
	vdW	–19.30 ± 0.41	–0.10 ± 0.02
6	electrostatic	597.19 ± 62.93	623.55 ± 79.22
	vdW	–1.26 ± 0.55	–0.89 ± 0.72
7	electrostatic	642.79 ± 163.80	588.00 ± 54.41
	vdW	–5.02 ± 0.73	–0.05 ± 0.02
8	electrostatic	643.29 ± 108.27	608.28 ± 55.32
	vdW	–1.29 ± 0.11	–0.12 ± 0.05
9	electrostatic	594.41 ± 175.17	525.99 ± 31.21
	vdW	–17.51 ± 3.24	–0.04 ± 0.09
10	electrostatic	622.38 ± 129.67	646.73 ± 110.27
	vdW	–4.27 ± 2.79	–0.29 ± 0.05
**average**	**electrostatic**	**629.15±26.9**	**611.04±38.09**
	**vdW**	**–6.14±6.38**	**–0.20±0.24**

a The standard deviations are represented with average values.

In general, the electrostatic energy of the membrane–dodecamer complex is nearly equivalent to that of the membrane–fibril complex, which seems to stem from the fact that both systems are composed of the same amount of monomers and identical total charge. However, the interaction energy also depends on the configuration of the charged residues, and, as shown above, although the electrostatic interaction energies between Aβ and the membrane are the same for two complexes, the minimum distance between the dodecamer and the membrane is less than the distance between the fibril and the membrane. This indicates that the arrangement of charges in the dodecamer leads to a weaker electrostatic interaction energy with the membrane than the fibril at the same distance from the membrane. Similarly, the repulsion between the dodecamer and the membrane is weaker than the fibril if the distance between each of them and the membrane is the same, which explains why the dodecamer can get closer to the membrane. This is also confirmed by the surface charge distribution (Figure 4), which shows that the dodecamer has more positively charged regions than the fibril exposed on the surface.

Since the dodecamer is closer to the membrane than the fibril (Figure 3), its vdW interaction with the membrane is stronger than the fibril (Table 2). We calculated the non-bonded interaction energy for snapshots that have a minimum distance between Aβ and the membrane of less than 10 Å. Such snapshots occurred in all MD trajectories in the dodecamer case, but for the fibril, they were observed only in trajectories 2, 5, 6, 8, and 10 (Table S1). Similar to the results obtained for all snapshots, electrostatic interaction energy dominates over the vdW interaction. The average non-bonded energies (Table S1) are lower than those obtained using all the collected snapshots (Table 2), but the difference is not significant in the standard deviation ranges. This result suggests that only a small fraction of the protein molecules is located near the membrane surface.

Drolle et al.^38^ reported that the roughness of the Alzheimer’s D1 membrane model fluctuates in the presence of Aβ aggregation but does not increase over time as in a healthy membrane. The surface roughness is proportional to the size of the Aβ aggregate bound to the membrane. During the first hour of incubation, only small spherical oligomers appeared on the surface of the D1 membrane, which suggests that the interaction of Aβ with the membrane led to the formation of oligomers instead of a mature fibril at short timescales. Since we used the D1 model, our result on the interaction and distance between Aβ and the membrane appears to be consistent with the experiment of Drolle et al.^38^ in the sense that oligomers are more likely to occur near the membrane than fibrils.

### GM1 Controls the Electrostatic Interaction between Aβ and the Membrane

To better understand the molecular mechanism underlying the Aβ–membrane stability, we evaluated the contribution of different groups of lipid molecules to the interaction energy between Aβ and the membrane. For both the dodecamer (Table S2) and the fibril (Table S3), DPPC, POPC, and PSM molecules have the attractive interaction with Aβ, while CHL1 and GM1 are pushed away from them due to the repulsive interaction. The contribution of GM1 prevails over other membrane components. The importance of GM1 in the Aβ–membrane interaction has also been demonstrated for another membrane model.^42^

**Figure 5:**
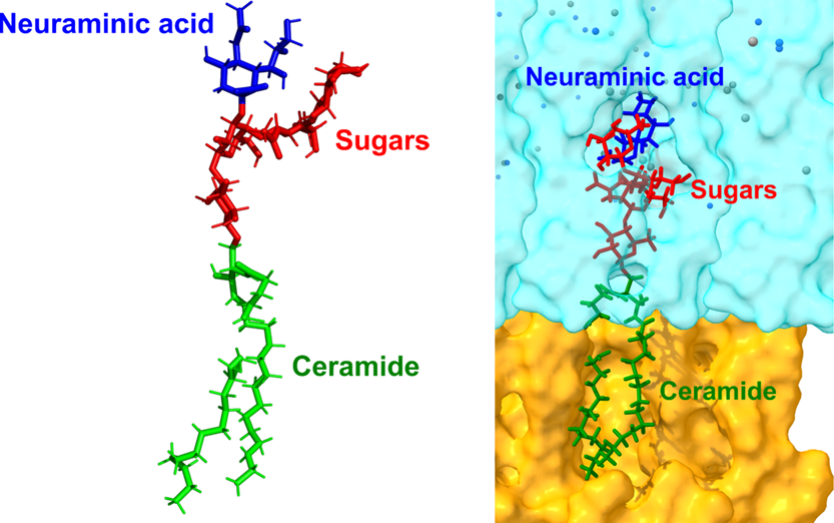
(Left) The GM1 molecules are composed of neuraminic acid, ceramide, and sugar. (Right) GM1 molecule in a membrane–solution system, water molecules are shown in transparent cyan, other lipids in yellow, and spheres refer to ions.

The GM1 molecule consists of three groups, including ceramide, neuraminic acid, and sugar (Figure 5). A detailed analysis showed that neuraminic acid and sugar groups equally contribute to the repulsive interaction with Aβ, while ceramide contributes to the attractive interaction (Tables S4 and S5). Thus, since the vdW interaction is weak, the neuraminic acid and sugar groups, which are exposed to the solution (Figure 5), control the interaction between Aβ and the membrane.

### Electrostatic Interaction Energy of Fibrils Is Stronger than That of Dodecamer

We calculated the inter-chain interaction energy for the dodecamer and fibrils; that is, the membrane–Aβ interaction was not taken into account. In both cases, the vdW interaction prevails over the electrostatic one (Table 3). The electrostatic energy in the fibril is significantly lower than that in the dodecamer, but in terms of vdW energy, the difference between them is small. Thus, the fibril is more stable than the dodecamer, which is reasonable because the fibrils are more structured than the oligomers. As shown before,^40^ monomers arrange their configuration in the fibril to minimize the electrostatic interaction energy to a greater extent than oligomers.

**Table 3:** Inter-chain Interaction Energy (kcal/mol) of the Aβ42 Dodecamer and Fibril^a^

trajectory	energy	Aβ42 dodecamer	Aβ42 fibril
1	electrostatic	–630.04 ± 85.21	–1299.30 ± 167.88
	vdW	–4095.45 ± 116.96	–4423.45 ± 190.66
2	electrostatic	–243.23 ± 75.45	–1176.58 ± 156.80
	vdW	–3912.34 ± 141.21	–4345.51 ± 221.94
3	electrostatic	–209.18 ± 89.42	–1785.32 ± 188.27
	vdW	–3944.63 ± 210.97	–4422.17 ± 252.35
4	electrostatic	127.45 ± 39.43	–1988.37 ± 193.48
	vdW	–4228.77 ± 91.69	–4411.70 ± 228.32
5	electrostatic	–349.88 ± 112.27	–1480.30 ± 113.60
	vdW	–3960.53 ± 161.00	–4478.78 ± 218.33
6	electrostatic	–510.96 ± 148.78	–1801.05 ± 190.70
	vdW	–4020.03 ± 130.79	–4475.35 ± 181.15
7	electrostatic	–188.74 ± 82.51	–2171.81 ± 155.95
	vdW	–3893.65 ± 141.69	–4234.57 ± 320.55
8	electrostatic	227.96 ± 51.77	–1794.86 ± 190.98
	vdW	–4085.98 ± 123.62	–4399.98 ± 200.85
9	electrostatic	–167.03 ± 65.59	–1276.56 ± 144.74
	vdW	–4044.03 ± 117.37	–4128.11 ± 278.22
10	electrostatic	–612.28 ± 97.63	–2261.22 ± 194.15
	vdW	–4059.52 ± 153.319	–4407.81 ± 185.33
**average**	**electrostatic**	**–255.59±85.83**	**–1703.54±114.42**
	**vdW**	**–4024.46±30.38**	**–4372.74±33.16**

a The membrane–Aβ interaction was not taken into account. The standard deviations are represented with average values.

### Secondary Structures: Dodecamer Is Richer in Helix than in β

We first recall the results previously obtained for the Aβ42 monomer. Using the CHARMM 36 m force field, Krupa et al. obtained β ≈ 15% and α ≈ 2% for the Aβ42 monomer in solution, which is consistent with the experiment.^72,73^ With the AMBER 14SB force field and TIP3P water model, it was shown that in the presence of the DMPC bilayer membrane, the Aβ42 monomer has β ≈ 8% and α ≈ 11%.^32^ Although it is difficult to compare the results obtained by different force fields, it is clear that the penetration of the monomer into the membrane increases the helix content, and this behavior is consistent with the NMR experiment^74^ and previous simulations.^75^ The interaction with the lipid tails was shown to allow Aβ to form a stable helical structure.^76^

We calculated the secondary structure of the peptides when they have contact with the membrane (Tables S6 and S7). For the Aβ42 dodecamer, the turn and coil dominate, and the helix content α (25.3%) is higher than the β content β (4.6%). The β content of Aβ42 dodecamer in this work is lower than the experimental estimate.^77^ The difference may be related to different types of membranes used in silico and in vitro (DOPS + POPE) experiments. More importantly, within the error bars, the secondary structure coincides with that of the starting structure used in the Aβ–membrane simulation (Table S6), which means that the secondary structure was mainly predetermined by the choice of the starting structure.

For fibrils, the β strand content is rich (β = 33%), which means that the fibrillar structure is preserved in the presence of the membrane due to the weak interaction between them. The helix structure was poorly populated (α ≈ 1%) because the fibril did not come close to the membrane. As in the dodecamer case, the secondary structure was driven by the choice of the starting structure (Table S7). Although the total populations of β and helix domains of the dodecamer and fibril are similar, the dodecamer is more flexible than the fibril in the arrangement of monomers, leading to reduced electrostatic repulsion from the membrane. This is because, as shown by experiment, the β requires global unfolding to swap, while the helix can be freely swapped locally.^78^ In the fibril, a rigid β sheet structure, which minimizes the electrostatic repulsion between monomers, prevents the structural rearrangement to reduce the repulsive interaction with the membrane. Consequently, it is easier for the dodecamer to approach the membrane than the mature fibril.

### Aβ Increases the Area Compressibility Modulus of the Membrane but Leaves the Bending Modulus Almost Unchanged

We calculated the bending modulus of the membrane in three cases, without Aβ, with dodecamer and fibril (Table 4). The presence of dodecamer and fibril insignificantly changes the bending modulus, which for three cases is about 10.1 × 10^–20^ J/m. This value is similar to other lipid raft membrane models.^79^ Thus, at the timescales used in our simulations, the Aβ42 dodecamer and fibril have negligible effect on the membrane bending modulus.

**Table 4:** Bending Modulus *K*_C_, Area Compressibility Modulus *K*_A_, Area per Lipid *A*, Membrane Thickness *d* of the Phosphate Group, and Lateral Diffusion Coefficient *D* of Lipids^a^

system	Aβ	*K*_C_ (10^–20^ J/m)	*K*_A_ (dyn/cm)	*A* (Å^2^)	*d* (Å)	*D* (10^–7^ cm^2^/s)
without protein		10.12	235.99	52.78 ± 0.48	41.17 ± 0.28	2.16 ± 0.12
1	dodecamer	10.12	309.06	52.69 ± 0.42	41.20 ± 0.25	1.67 ± 0.13
	fibril	10.22	236.92	52.66 ± 0.48	41.23 ± 0.28	1.15 ± 0.15
2	dodecamer	10.08	304.40	52.68 ± 0.42	41.17 ± 0.25	2.42 ± 0.24
	fibril	10.14	276.79	52.73 ± 0.45	41.17 ± 0.26	2.10 ± 0.49
3	dodecamer	10.11	325.52	52.64 ± 0.41	41.23 ± 0.25	0.97 ± 0.12
	fibril	10.15	240.59	52.71 ± 0.48	41.19 ± 0.29	2.84 ± 0.23
4	dodecamer	10.10	272.75	52.62 ± 0.45	41.24 ± 0.28	4.32 ± 0.61
	fibril	10.04	238.32	52.69 ± 0.48	41.06 ± 0.32	2.01 ± 0.12
5	dodecamer	10.08	276.45	52.60 ± 0.44	41.27 ± 0.27	1.45 ± 0.88
	fibril	10.20	302.89	52.68 ± 0.42	41.17 ± 0.28	2.39 ± 0.21
6	dodecamer	10.17	266.04	52.65 ± 0.45	41.20 ± 0.25	1.75 ± 0.21
	fibril	10.30	296.19	52.58 ± 0.43	41.22 ± 0.28	0.61 ± 0.05
7	dodecamer	10.12	264.81	52.71 ± 0.45	41.18 ± 0.27	1.17 ± 0.78
	fibril	10.06	235.44	52.76 ± 0.48	41.09 ± 0.31	2.89 ± 0.18
8	dodecamer	10.11	216.58	52.73 ± 0.50	41.15 ± 0.28	1.73 ± 0.29
	fibril	10.09	269.55	52.62 ± 0.45	41.21 ± 0.27	4.70 ± 0.68
9	dodecamer	10.07	252.98	52.72 ± 0.46	41.18 ± 0.25	2.62 ± 0.89
	fibril	10.11	247.51	52.75 ± 0.47	41.26 ± 0.43	1.24 ± 0.32
10	dodecamer	10.15	269.69	52.68 ± 0.45	41.18 ± 0.26	1.61 ± 0.16
	fibril	10.07	271.48	52.67 ± 0.45	41.19 ± 0.27	0.68 ± 0.12
average	dodecamer	10.11 ± 0.03	275.83 ± 29.47	52.67 ± 0.04	41.20 ± 0.03	1.97 ± 0.29
	fibril	10.14 ± 0.08	256.17 ± 27.06	52.69 ± 0.05	41.18 ± 0.06	2.06 ± 0.37

a The standard deviations are represented with average values.

On the contrary, the area compressibility modulus (*K*_A_) of the membrane depends on the systems being simulated (Table 4). In the absence of peptides, *K*_A_ is 235.99 dyn/cm, which falls within the range of experimental data of 216–244 dyn/cm reported for a membrane model consisting of 14–18% CHL1 and SOPC.^80^ In the presence of Aβ42 dodecamer and fibril, *K*_A_ increases to 275.83 ± 29.47 and 261.57 ± 24.02 dyn/cm, respectively. Thus, the dodecamer alters *K*_A_, which was calculated from the fluctuation of the area per lipid, more strongly than the fibril. This result is reasonable, as the dodecamer approaches the membrane closer than the fibril (Figure 3), causing less fluctuations of the membrane surface.

### Aβ Does Not Change the Area per Lipid *A*

The areas per lipid *A* are practically identical in the presence and absence of peptides (Table 4). For all systems, the area per lipid is about 52.7 Å^2^ (Table 4), which is less than the value for a single lipid membrane such as DPPC (64.2 Å^2^) and POPC (68.3 Å^2^).^81^ The presence of cholesterol results in a lower *A* value in the POPC/cholesterol (50:50) membrane (45.1 ± 0.9 Å^2^)^82^ than a pure POPC membrane, and cholesterol enhances lipid packing in the raft membrane model.^81^ Our *A* value is reasonable as it is close to 53.0 ± 0.7 Å^2^ of the POPC/cholesterol (80:20) membrane with a cholesterol ratio similar to our model (18%).^83^

### Lipid Molecules in Our Model Are More Mobile than in a Model with Two Types of Lipids

In the absence of Aβ, the diffusion coefficient *D* is 2.16 ± 0.12 (10^–7^ cm^2^/s) (Table 4), which is higher than *D* ≈ 1.7 (10^–7^ cm^2^/s) obtained in the experiment for DOPC/cholesterol and POPC/cholesterol (80:20) membranes.^84^ For DOPC/cholesterol (80:20) and SM/cholesterol (80:20), the *D* value is about 1.5 and 0.5 (10^–7^ cm^2^/s), respectively.^85^ Thus, our *D* value is higher than that of membrane models consisting of two types of lipids, which is probably due to the fact that our model has more types of lipids, and this leads to a higher mobility of lipid molecules.

### Dodecamer Impacts Lipid Arrangement More Strongly than Fibril

**Figure 6:**
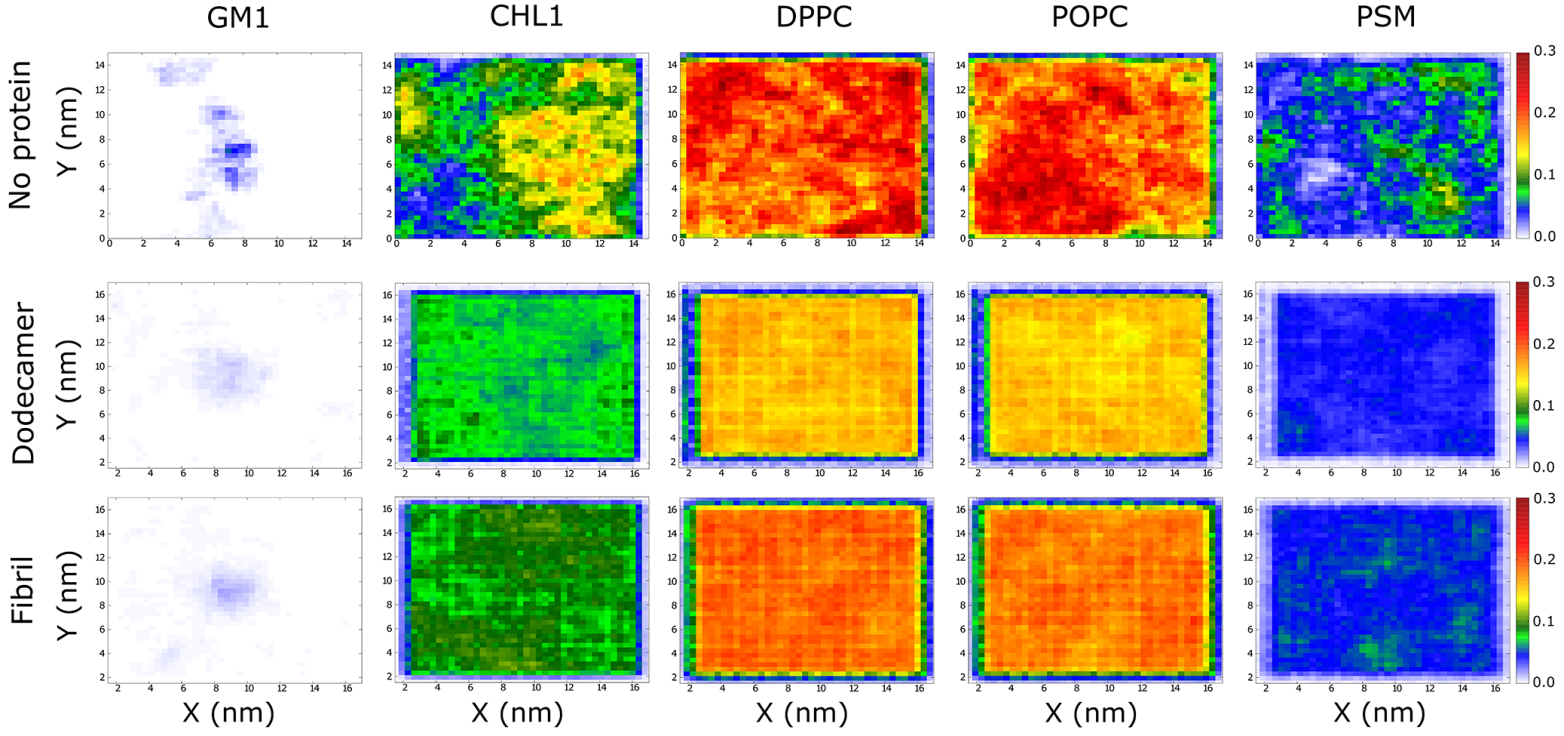
Distribution of lipids. The results were obtained by averaging over all trajectories for the membrane–dodecamer and membrane–fibril complexes.

Even without Aβ, the density of lipid molecules is heterogeneous (Figure 6). Cholesterol (CHL1) molecules are concentrated in three distinct regions, among which two islets of high density (yellow-orange) are surrounded by a blue-green region of low density. In the case of GM1 and PSM, the lipid distribution shows clearly high-population regions (Figure 6), but, unlike CHL1, they are small and scattered. For DPPC and POPC, we observed large areas of high lipid content, but areas of high DPPC density interspersed with areas of low POPC density and vice versa, which indicates that these lipid molecules promote uniform distribution.

With the dodecamer, the distribution of CHL1 is more uniform than in the case of a membrane without Aβ (Figure 6). This type of distribution is also true for other lipids. DPPC and POPC do not have distinct regions as in the absence of a dodecamer. In particular, in trajectories 4, 5, and 6, CHL1 molecules are distributed in regions with different densities (Figures S6 and S7). Areas of high population are sparse on the membrane surface, which differs from the distribution of CHL1 molecules without peptides. In other MD runs, CHL1 molecules are evenly distributed without high-density areas.

As in the case without Aβ, for DPPC and POPC, the distribution is uniform, except for trajectories 4, 5, and 6, where the difference in lipid densities between the regions of high and low population is pronounced (Figures S6 and S7). However, after averaging over all MD runs, the distribution of these lipids becomes relatively homogeneous (Figure 6). The distribution of PSM is noticeably heterogeneous. Specially, in trajectories 4, 5, and 6, we have small areas with a higher density than the rest, while in other trajectories, the difference in density is less (Figures S7 and S8). These results indicate that the presence of dodecamer alters the distribution of lipid molecules, causing them to be distributed more evenly than the membrane alone.

In the presence of fibril, on average, the distribution of lipids is more uniform than in their absence (Figure 5). Although there are no areas with a clearly high population, as in the case of membrane only, the CHL1 distribution is still divided into large areas with clearly different populations. For DPPC, POPC, and PSM, there exist only small regions with a relatively high population compared to other regions, which is different from the pure membrane. Consequently, the effect of dodecamer on lipid distribution is more pronounced than fibrils, even when the distribution is averaged over all MD trajectories. In trajectories 1–9, CHL1 molecules are concentrated in high-density areas (Figures S9 and S10). This formation of regions with different densities is analogous to the case of only membrane. In run 10, the distribution of CHL1 is uniform, which is similar to runs 1–3, 7–10 of the dodecamer–membrane complex. Like CHL1, in trajectories 1–9, DPPC, POPC, and PSM lipids have regions of much higher density than other regions (Figures S8 and S9). In trajectory 10, the distribution of DPPC, POPC, and PSM is more uniform than in other systems, which is similar to trajectories 1–3 and 7–10 of the dodecamer (Figures S6–S9). Consequently, the fibril has a weaker effect on lipid distribution than the dodecamer. Lipid molecules are rearranged on the membrane surface in the presence of the dodecamer, which leads to a uniform distribution, while the fibril only shuffles the regions of different densities on the membrane surface. GM1 molecules in the absence of Aβ try to cluster tightly, while in the presence of peptides, their interaction with the membrane leads to a more scattered distribution of GM1 (Figure 6). The differences in the distribution of GM1 for the dodecamer and fibrils are insignificant.

### Aβ Has Minor Effect on the Radial Distribution Function of Cholesterol and Lipid Molecules

**Figure 7:**
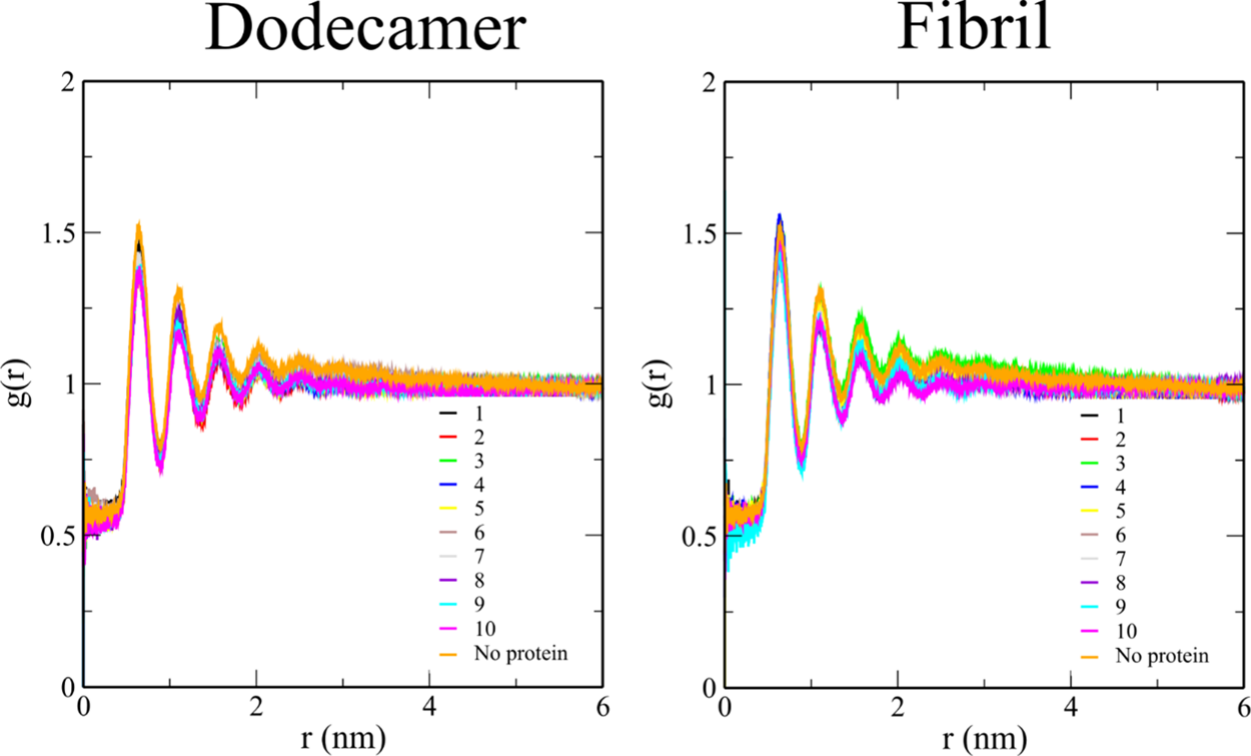
Radial distribution function *g*(*r*) of cholesterol molecules in membrane–dodecamer and membrane–fibril systems. The Aβ-free case is shown in orange. The reference point is the center of mass of cholesterol residues.

The radial distribution functions of cholesterol molecules in the presence of dodecamer and fibril are shown in Figure 7. All systems have similar curves with the first three peaks at 0.6, 1.1, and 1.5 Å. In the presence of dodecamer and fibril, the *g*(*r*) value at the peaks changes slightly, but the position of the peaks is preserved (Figure 7), indicating that the effect of Aβ on cholesterol *g*(*r*) is insignificant. Like CHL1, the distribution function *g*(*r*) of other lipids does not change much in the presence of Aβ (data not shown). This is because both the dodecamer and the fibril do not penetrate the membrane deeply.

### Aβ Has Little Effect on the Acyl Chain Order Parameter

**Figure 8:**
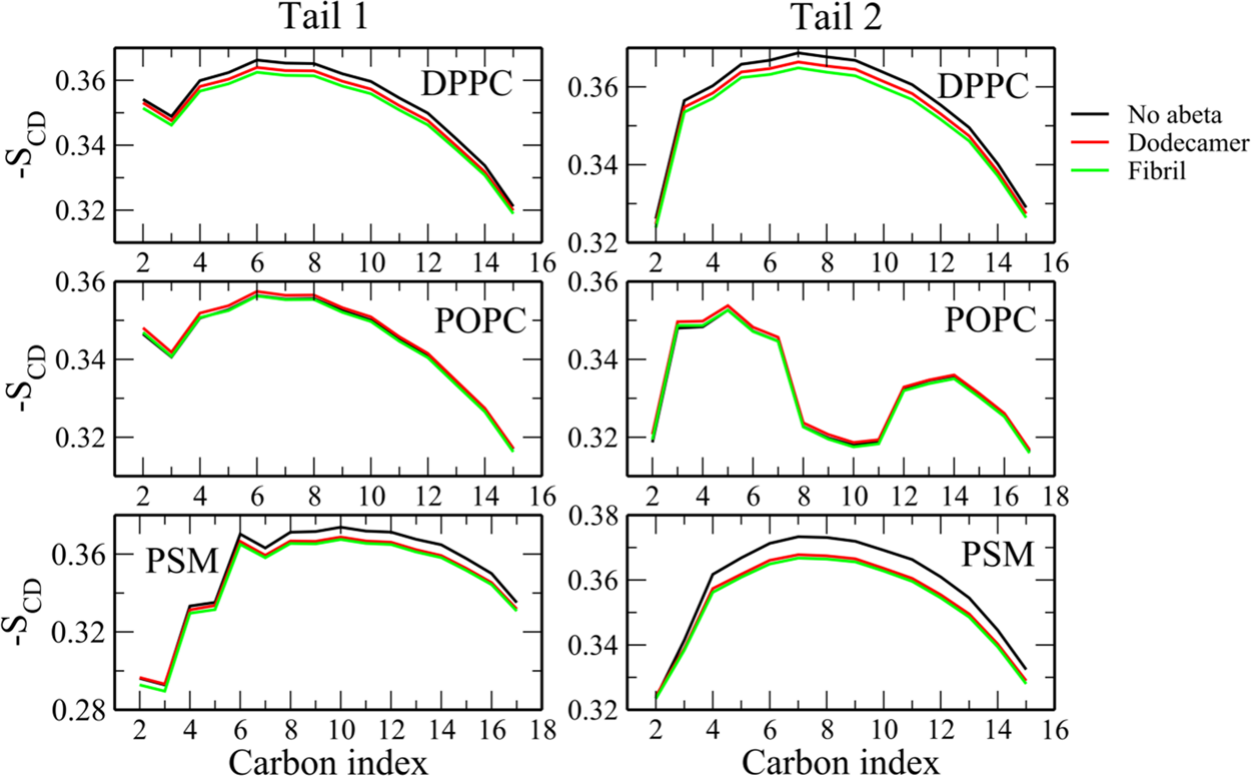
Tail order parameters of lipid molecules in the absence and presence of Aβ. Results were averaged over 10 MD trajectories.

We can show that the −*S*_CD_ order parameters of carbon atoms in the tails are about 0.3 (Figures S10 and S11), which is consistent with the previous simulation results for DPPC/Chol and PSM/Chol.^86^ In the presence of dodecamer, −*S*_CD_ of DPPC and PSM is slightly reduced in all MD runs (Figure S11). For POPC, the presence of dodecamer has no visible effect. With the fibril, this order parameter depends on MD runs (Figure S12), but after averaging over all trajectories, the order parameters of carbon in tails of lipid molecules remain almost unchanged (Figure 8). Thus, the effect of both the fibril and the dodecamer is weak (Figure 8) since Aβ can only approach the membrane surface but not penetrate it.

### Aβ Changes the Distribution of the Membrane Thickness

**Figure 9:**
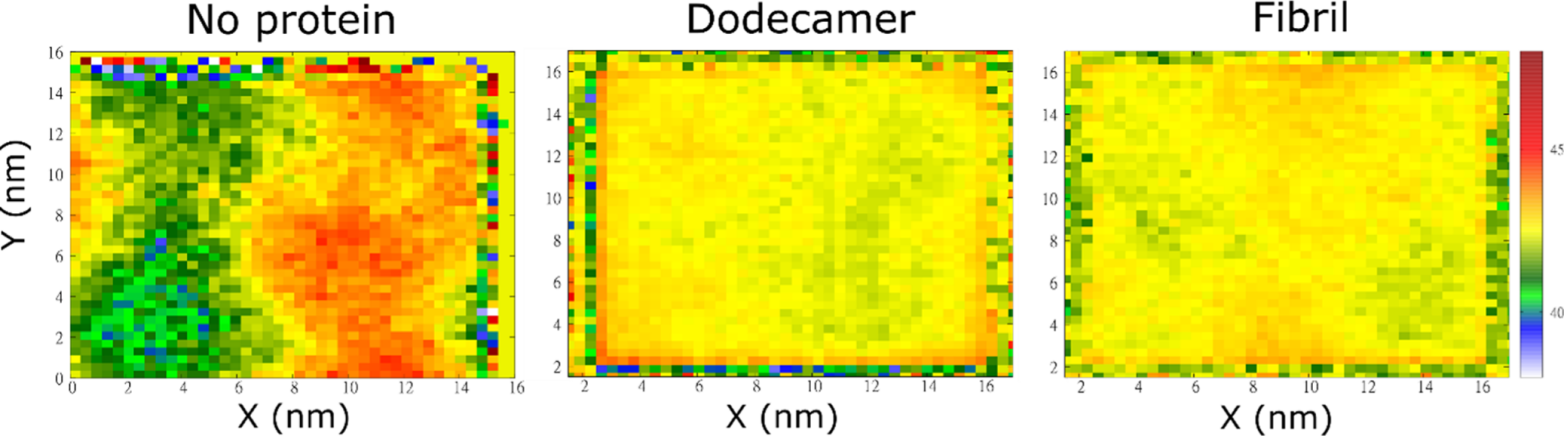
Distribution of the membrane thickness for the membrane alone, membrane–dodecamer, and membrane–fibril complexes. The results were averaged over all MD trajectories.

Using phosphorus atoms, we calculated the membrane thickness. Without Aβ, the distribution of membrane thickness has two regions with different values (Figure 9), and interestingly, the position of these regions correlates with the distribution of CHL1 (compare Figure 9 with Figure 6). The high-density region of CHL1 is thicker than the other regions, which is consistent with the experiment.^87^

In MD runs 6 and 10, the membrane–dodecamer complex has distinctly thick regions, but the thickness distribution is relatively uniform in other runs (Figure S13). Similar to the case of membrane only, the position of the thick regions in runs 6 and 10 correlates with the position of the high CHL1 density regions (Figure 6). Averaging over 10 trajectories shows that the presence of dodecamer significantly alters the thickness distribution (Figure 9), as the sharp difference in thickness between areas is eliminated, making the distribution even.

For the membrane–fibril complex, we observed bulky regions with a thickness of about 45 Å in trajectories 1, 2, 4, 5, 6, 7, 8, 9, and 10 (Figure S14). Similar to the only membrane case, the thickness is distributed heterogeneously, but averaging over 10 trajectories, we obtained a uniform distribution (Figure 9). Therefore, the Aβ–membrane interaction makes the thickness distribution more homogeneous.

Although the presence of Aβ peptides changes the membrane thickness distribution, the average thickness *d* is negligibly affected. Considering only P atoms, we obtained *d* ≈ 41.1 Å for three systems (Table 4), which is consistent with the simulation results for POPC/cholesterol (42.8 ± 0.5 Å)^83^ and DPPC/cholesterol membrane (39.4–44.8 Å).^88^

### Cracks of the Membrane Surface

We defined a membrane crack as a space within the top and bottom of the bilayer that is not occupied by a single atom of the membrane. An atom is represented by a sphere with a vdW radius. The depth of the surface crack was calculated as the distance from the top lipid layer to the bottom of the crack (Figure S15).

**Figure 10:**
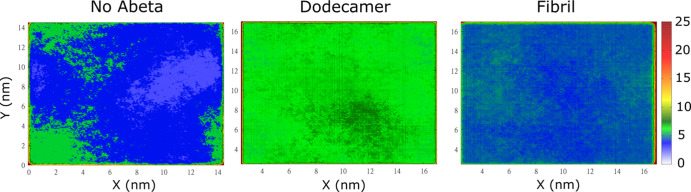
Distribution of the depth of cracks on the membrane surface for the membrane alone, membrane–dodecamer, and membrane–fibril complexes. The results were averaged over all MD trajectories.

Without Aβ, the membrane has only tiny surface cracks (Figure 10) that arise from fluctuations of the lipid heads, suggesting that the membrane structure is very stable without spontaneous structural defects. In the presence of a dodecamer, the distribution of cracks depends on MD runs (Figure S16). After averaging over all MD trajectories, we see that not only the depth of the cracks increases but also their number (Figure 10), which indicates that the dodecamer softens the membrane. However, there is no crack running through the membrane (i.e., there is no channel), which indicates that the dodecamer affects the rigidity of the membrane surface, creating more space between the lipid heads, but not the acyl chains. This is reasonable since the dodecamer approaches only the membrane surface, having mild effect on the stabilization of the membrane structure.

**Figure 11:**
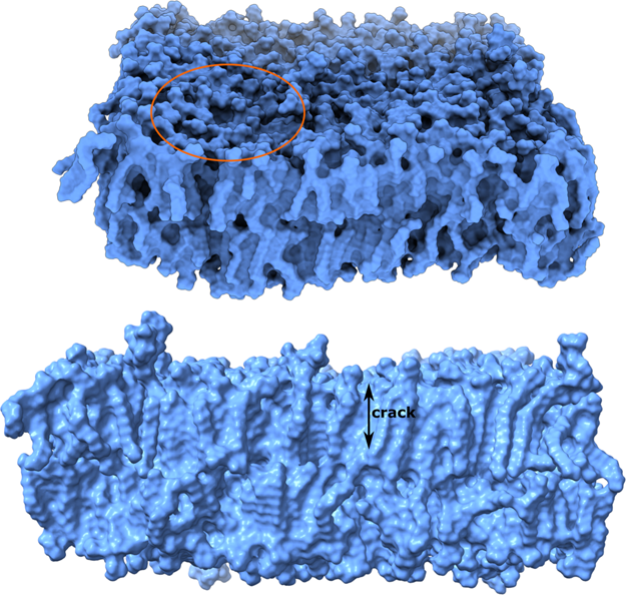
Position of the deepest crack observed in the dodecamer simulation (top). This crack spans almost half of the membrane thickness (bottom).

In run 5 for the fibril case, the cracks are deeper than the Aβ-free case, but in other runs, the difference is smaller (Figure S16). The area of shallow cracks is larger than in the dodecamer case (Figure 10). Averaging over 10 MD runs, we obtain the crack depths 7.26 ± 0.65 and 5.12 ± 0.72 Å for the dodecamer and fibrils, respectively. The deepest crack of 17.07 Å observed in our simulations for the dodecamer covers almost half of the membrane thickness (Figure 11). Thus, while our short simulations do not allow pore formation, the results suggest that oligomers are better than fibrils at promoting those structural fluctuations that can eventually facilitate channel formation.

## Conclusions

Using MD simulation, we were able to reproduce the experimental area compressibility modulus for a realistic neuron membrane. We predict that the presence of Aβ oligomers at the membrane surface makes the membrane more rigid, leading to an increase in this modulus. We have shown that, in accordance with the experiment, the high-density CHL1 regions are thicker than others. The presence of Aβ significantly alters the distribution of lipid molecules in the multi-lipid bilayer model.

Due to the difference in the charge distribution on the surface, the dodecamer can approach the membrane closer than the fibril disturbing the membrane to a greater extent. Although we did not observe channel structures due to the short simulation timescale, cracks on the membrane surface are more pronounced and populated in the dodecamer case than in the case of fibril. This may support the experimental fact that oligomers are more toxic to cells than mature fibrils. We have shown that neuraminic acid and sugar groups of the ganglioside GM1 lipid, which are exposed to water, control the interaction between Aβ and the membrane.

The bacterial membranes comprise multiple lipid species;^89^ the major lipids are negatively charged lipids, while the minor lipids can be positively charged. Therefore, the cell membrane charge of many bacteria is negative,^90^ which is similar to the membrane model studied in this work. From this perspective, we expect that Aβ affects the bacterial membrane in the same way as it does with the present Alzheimer membrane model. In other words, our study shed some light on Aβ binding to microbial surfaces, which are related to their antimicrobial properties.^91^

## Supporting Information Available

Interaction energy between the Aβ42 dodecamer and the fibril with the membrane when the minimum distance between them is equal to or less than 10 Å; interaction energy between different lipid groups and dodecamer; interaction energy between different lipid groups and fibril; interaction energy of GM1 groups with dodecamer; interaction energy (kcal/mol) of GM1 groups with fibril; secondary structures of the dodecamer; secondary structures of the mature fibril; initial structures used in MD simulations for membrane–dodecamer and membrane–fibril complexes; definition of the θ angle between the C–H bond vector and the *z*-axis; time dependence of the rmsd of C-alpha atoms of dodecamer and mature fibril in solution; structural properties of the Aβ dodecamer and fibril: time dependence of rmsd, *R*_g_, number of contacts between Aβ chains and SASA in the production run of 800 ns; average minimum distance between the dodecamer and fibril and the membrane center along the *z*-axis; snapshot showing the touch of the Aβ42 dodecamer to the membrane surface; distribution of lipid molecules in trajectories 1–5 of the membrane–dodecamer complex; distribution of lipid molecules in trajectories 6–10 of the membrane–dodecamer complex; distribution of lipids in trajectories 1–5 of the membrane–fibril complex; distribution of lipids in trajectories 6–10 of the membrane–fibril complex; tail order parameters of lipid molecules in the presence of Aβ42 dodecamer; tail order parameters of lipid molecules in the presence of Aβ42 fibril; distribution of the membrane thickness in the presence of Aβ42 dodecamer; distribution of the membrane thickness in the presence of Aβ42 fibril; definition of the crack depth; and distribution of crack depths on the membrane surface (PDF)
