# Supplementary material for: Amyloid β Dodecamer Disrupts the Neuronal Membrane More Strongly than the Mature Fibril: Understanding the Role of Oligomers in Neurotoxicity
Source: J Phys Chem B. 2022 May 17;126(20):3659–72. doi: 10.1021/acs.jpcb.2c01769 (PMC9150093; doi:10.1021/acs.jpcb.2c01769)
Supplement: Supplementary file 1 — jp2c01769_si_001.pdf [file jp2c01769_si_001.pdf]

Amyloid Beta Dodecamer Disrupts the Neuronal Membrane More Strongly than Mature Fibril: Understanding the Role of Oligomers in Neurotoxicity

Hoang Linh Nguyen,[1,2,3] Huynh Quang Linh,[2,3] , Pawel Krupa[4], Giovanni La Penna[5,6], and Mai Suan Li[4,*]

[1]Institute for Computational Science and Technology, SBI Building, Quang Trung Software City, Tan Chanh Hiep Ward, District12, Ho Chi Minh City 700000, Vietnam
[2]Ho Chi Minh City University of Technology (HCMUT), Ho Chi Minh City 700000, Vietnam
[3]Vietnam National University, Ho Chi Minh City 700000, Vietnam

[4]Institute of Physics, Polish Academy of Sciences, Al. Lotnikow 32/46, 02-668 Warsaw, Poland

[5]National Research Council of Italy (CNR), Institute for Chemistry of Organometallic Compounds (ICCOM), 50019 Florence, Italy
[6]National Institute for Nuclear Physics (INFN), Section of Roma-Tor Vergata

E-mail: masli@ifpan.edu.pl

SUPPORTTING INFORMATION

Table S1: Interaction energy (kcal / mol) between the Aβ42 dodecamer and the fibril with the membrane when the minimum distance between them is equal to or less than 10 Å. The standard deviations are represented with average values. In the fibril case the minimum distance always exceeds 10 Å in trajectories 1, 3, 4, 7 and 9.

Trajectory	Energy	Aβ42 dodecamer	Aβ42 fibril
1	Electrostatic	602.91 ± 60.48	
	van der Waals	-5.06 ± 3.12	
2	Electrostatic	616.40 ± 75.96	672.73 ± 90.11
	van der Waals	-8.24 ± 2.28	-2.80 ± 0.66
3	Electrostatic	667.68 ± 75.68	
	van der Waals	-3.92 ± 1.76	
4	Electrostatic	608.03 ± 47.86	
	van der Waals	-6.94 ± 3.36	
5	Electrostatic	564.21 ± 77.49	651.83 ± 73.56
	van der Waals	-23.28 ± 2.89	-1.44 ± 0.50
6	Electrostatic	577.87 ± 86.78	538.48 ± 52.48
	van der Waals	-3.69 ± 2.08	-5.22 ± 1.38
7	Electrostatic	593.89 ± 93.98	
	van der Waals	-7.02 ± 3.99	
8	Electrostatic	629.88 ± 84.81	544.67 ± 29.82
	van der Waals	-3.56 ± 1.27	-0.82 ± 0.34
9	Electrostatic	583.27 ± 69.33	
	van der Waals	-22.60 ± 2.71	
10	Electrostatic	575.52 ± 75.84	631.58 ± 21.11
	van der Waals	-7.39 ± 2.43	-3.19 ± 1.72
**Average**	**Electrostatic**	**601.97 ± 29.09**	**607.86 ± 55.69**
	**van der Waals**	**-9.17 ± 7.06**	**-2.69 ± 1.53**

Table S2: Interaction energy (kcal/mol) between different lipid groups and dodecamer.

System		CHL1	GM1	DPPC	POPC	PSM
1	Electrostatic	4.18 ± 1.24	885.71 ± 73.97	-120.36 ± 21.77	-113.65 ± 11.31	-21.37 ± 9.35
	vdW	-0.26 ± 0.01	-0.28 ± 0.19	-0.42 ± 0.39	-0.41 ± 0.34	-0.31 ± 0.19
2	Electrostatic	5.47 ± 1.41	940.57 ± 71.72	-143.53 ± 19.25	-124.45 ± 9.34	-28.11 ± 7.40
	vdW	-0.71 ± 0.02	-0.78 ± 0.31	-1.13 ± 0.78	-1.06 ± 0.65	-0.87 ± 0.45
3	Electrostatic	2.14 ± 0.98	867.75 ± 71.69	-89.48 ± 12.37	-87.70 ± 6.61	-17.39 ± 11.75
	vdW	-0.09 ± 0.01	-0.13 ± 0.06	-0.14 ± 0.04	-0.15 ± 0.08	-0.11 ± 0.04
4	Electrostatic	4.32 ± 2.15	835.46 ± 65.14	-92.96 ± 18.58	-86.37 ± 10.41	-16.15 ± 8.67
	vdW	-1.11 ± 0.01	-1.19 ± 0.34	-1.24 ± 0.46	-1.21 ± 0.29	-1.13 ± 0.09
5	Electrostatic	8.27 ± 3.41	976.31 ± 77.36	-182.29 ± 18.51	-176.97 ± 13.81	-37.89 ± 5.62
	vdW	-3.39 ± 0.03	-3.61 ± 0.69	-4.34 ± 1.36	-4.29 ± 1.31	-3.66 ± 0.62
6	Electrostatic	5.28 ± 2.55	799.59 ± 78.08	-88.63 ± 11.19	-98.88 ± 10.85	-20.18 ± 11.33
	vdW	-0.20 ± 0.08	-0.22 ± 0.14	-0.30 ± 0.20	-0.32 ± 0.06	-0.23 ± 0.13
7	Electrostatic	0.55 ± 0.44	889.87 ± 77.47	-116.47 ± 10.62	-106.40 ± 9.85	-24.76 ± 12.73
	vdW	-0.86 ± 0.13	-0.92 ± 0.39	-1.12 ± 0.58	-1.15 ± 0.61	-0.97 ± 0.38
8	Electrostatic	2.40 ± 0.52	804.47 ± 74.26	-76.75 ± 14.98	-73.90 ± 6.78	-12.92 ± 3.31
	vdW	-0.25 ± 0.01	-0.25 ± 0.07	-0.26 ± 0.03	-0.29 ± 0.24	-0.24 ± 0.01
9	Electrostatic	4.56 ± 2.78	1007.50 ± 98.65	-192.27 ± 26.36	-188.93 ± 15.50	-36.46 ± 7.99
	vdW	-2.99 ± 0.25	-3.39 ± 0.93	-3.86 ± 0.97	-3.99 ± 1.25	-3.26 ± 0.47
10	Electrostatic	1.99 ± 0.51	966.70 ± 69.41	-117.77 ± 14.96	-106.94 ± 14.72	-21.60 ± 11.12
	vdW	-0.70 ± 0.22	-0.71 ± 0.16	-1.08 ± 0.78	-0.93 ± 0.43	-0.85 ± 0.39
average	Electrostatic	3.92 ± 2.10	887.39 ± 65.77	-122.05 ± 37.61	-116.42 ± 36.09	-23.69 ± 7.87
	vdW	-1.06 ± 1.01	-1.15 ± 1.12	-1.39 ± 1.21	-1.38 ± 1.13	-1.16 ± 1.22

Table S3: Interaction energy (kcal/mol) between different lipid groups and fibril.

System		CHL1	GM1	DPPC	POPC	PSM
1	Electrostatic	6.15 ± 2.81	861.68 ± 92.81	-90.89 ± 15.97	-89.13 ± 13.21	-12.10 ± 9.98
	vdW	-0.03 ± 0.01	-0.03 ± 0.01	-0.04 ± 0.02	-0.03 ± 0.01	-0.04 ± 0.01
2	Electrostatic	5.13 ± 2.38	787.34 ± 79.11	-77.16 ± 10.66	-69.67 ± 7.87	-12.33 ± 7.47
	vdW	-0.03 ± 0.01	-0.03 ± 0.01	-0.04 ± 0.01	0.02 ± 0.02	-0.03 ± 0.01
3	Electrostatic	5.54 ± 2.30	757.65 ± 74.72	-70.74 ± 14.94	-68.10 ± 14.04	-11.54 ± 5.66
	vdW	-0.01 ± 0.01	-0.01 ± 0.01	-0.01 ± 0.01	-0.01 ± 0.01	-0.01 ± 0.01
4	Electrostatic	3.73 ± 2.01	720.84 ± 74.33	66.48 ± 7.15	-60.60 ± 14.68	-11.30 ± 5.10
	vdW	-0.01 ± 0.01	-0.01 ± 0.01	-0.01 ± 0.01	-0.01 ± 0.01	-0.01 ± 0.01
5	Electrostatic	5.75 ± 1.94	748.42 ± 66.17	-69.41 ± 6.26	-63.68 ± 13.04	-11.35 ± 6.05
	vdW	-0.01 ± 0.01	-0.01 ± 0.01	-0.01 ± 0.02	-0.01 ± 0.02	-0.01 ± 0.01
6	Electrostatic	6.31 ± 2.66	831.55 ± 84.48	-102.62 ± 12.80	-94.52 ± 11.46	-17.17 ± 5.98
	vdW	-0.15 ± 0.01	-0.15 ± 0.01	-0.23 ± 0.30	-0.20 ± 0.15	-0.16 ± 0.05
7	Electrostatic	2.96 ± 1.81	702.93 ± 67.68	-54.25 ± 9.21	-56.36 ± 10.10	-7.29 ± 4.20
	vdW	-0.01 ± 0.01	-0.01 ± 0.01	-0.01 ± 0.01	-0.01 ± 0.01	-0.01 ± 0.01
8	Electrostatic	4.39 ± 1.86	766.39 ± 66.95	-75.29 ± 14.67	-71.40 ± 8.19	-15.83 ± 7.61
	vdW	-0.02 ± 0.01	-0.02 ± 0.01	-0.03 ± 0.01	-0.03 ± 0.01	-0.02 ± 0.04
9	Electrostatic	-2.92 ± 1.05	669.40 ± 82.51	-64.84 ± 14.95	-60.79 ± 5.29	-14.85 ± 2.56
	vdW	-0.01 ± 0.01	-0.01 ± 0.01	-0.01 ± 0.01	-0.01 ± 0.01	-0.01 ± 0.01
10	Electrostatic	3.67 ± 0.88	829.08 ± 77.67	-85.94 ± 10.22	-86.29 ± 8.56	-13.88 ± 4.81
	vdW	-0.05 ± 0.01	-0.06 ± 0.02	-0.07 ± 0.02	-0.06 ± 0.02	-0.05 ± 0.01
average	Electrostatic	4.07 ± 2.56	767.53 ± 9.29	-75.76 ± 20.75	-72.05 ± 14.67	-12.75 ± 9.78
	vdW	-0.03 ± 0.03	-0.03 ± 0.03	-0.04 ± 0.03	-0.04 ± 0.03	-0.03 ± 0.01

Table S4: Interaction energy (kcal/mol) of GM1 groups with dodecamer.

		ceramide	Neuraminic acid	Sugar groups
1	electrostatic	-167.676 ± 16.625	558.658 ± 58.366	494.724 ± 83.757
	Van der Waals	-0.033 ± 0.005	-0.066 ± 0.026	-0.185 ± 0.035
2	electrostatic	-180.609 ± 16.898	585.093 ± 54.719	536.090 ± 72.791
	Van der Waals	-0.089 ± 0.043	-0.210 ± 0.065	-0.483 ± 0.096
3	electrostatic	-165.569 ± 13.573	533.592 ± 47.635	499.725 ± 72.954
	Van der Waals	-0.002 ± 0.002	-0.041 ± 0.036	-0.090 ± 0.067
4	electrostatic	-159.998 ± 13.551	520.338 ± 57.135	475.168 ± 69.417
	Van der Waals	-0.174 ± 0.013	-0.333 ± 0.092	-0.684 ± 0.339
5	electrostatic	-183.435 ± 15.222	610.349 ± 54.310	549.397 ± 68.990
	Van der Waals	-0.523 ± 0.021	-0.959 ± 0.560	-2.131 ± 1.016
6	electrostatic	-154.087 ± 16.145	502.878 ± 55.070	450.802 ± 87.821
	Van der Waals	-0.024 ± 0.003	-0.046 ± 0.013	-0.146 ± 0.016
7	electrostatic	-173.074 ± 17.889	556.557 ± 57.732	506.383 ± 75.731
	Van der Waals	-0.129 ± 0.009	-0.243 ± 0.074	-0.550 ± 0.089
8	electrostatic	-154.976 ± 15.776	506.542 ± 52.997	452.906 ± 70.559
	Van der Waals	-0.046 ± 0.002	-0.050 ± 0.045	-0.154 ± 0.021
9	electrostatic	-193.500 ± 23.099	630.904 ± 81.368	570.097 ± 90.676
	Van der Waals	-0.347 ± 0.046	-0.852 ± 0.606	-2.192 ± 1.211
10	electrostatic	-166.11 ± 15.099	541.919 ± 44.200	490.893 ± 96.930
	Van der Waals	-0.124 ± 0.004	-0.139 ± 0.071	-0.443 ± 0.036
average	electrostatic	-169.903 ± 16.387	554.683 ± 56.354	502.619 ± 83.962
	Van der Waals	-0.149 ± 0.015	-0.294 ± 0.067	-0.706 ± 0.069

Table S5: Interaction energy (kcal/mol) of GM1 groups with fibril.

		ceramide	Neuraminic acid	Sugar groups
1	electrostatic	-163.651 ± 17.803	525.701 ± 77.136	499.629 ± 66.210
	Van der Waals	-0.005 ± 0.001	-0.010 ± 0.004	-0.015 ± 0.010
2	electrostatic	-150.440 ± 15.574	476.090 ± 50.695	461.689 ± 67.665
	Van der Waals	-0.006 ± 0.001	-0.006 ± 0.001	-0.018 ± 0.002
3	electrostatic	-147.273 ± 17.112	473.926 ± 63.93	430.995 ± 62.559
	Van der Waals	-0.002 ± 0.001	-0.002 ± 0.002	-0.007 ± 0.005
4	electrostatic	-137.297 ± 14.659	440.201 ± 42.751	417.936 ± 59.854
	Van der Waals	-0.002 ± 0.001	-0.002 ± 0.001	-0.006 ± 0.003
5	electrostatic	-140.250 ± 11.796	459.558 ± 39.779	429.107 ± 60.488
	Van der Waals	-0.001 ± 0.001	-0.001 ± 0.001	-0.008 ± 0.005
6	electrostatic	-156.944 ± 15.574	524.089 ± 53.179	464.401 ± 88.438
	Van der Waals	-0.029 ± 0.001	-0.030 ± 0.005	-0.090 ± 0.028
7	electrostatic	-133.300 ± 11.965	431.247 ± 50.562	404.986 ± 64.221
	Van der Waals	-0.002 ± 0.001	-0.003 ± 0.001	-0.005 ± 0.001
8	electrostatic	-144.821 ± 11.664	470.655 ± 41.105	440.557 ± 71.602
	Van der Waals	-0.004 ± 0.001	-0.004 ± 0.003	-0.012 ± 0.002
9	electrostatic	-129.870 ± 17.626	426.289 ± 68.820	372.977 ± 59.745
	Van der Waals	-0.002 ± 0.001	-0.001 ± 0.001	-0.008 ± 0.002
10	electrostatic	-153.761 ± 18.189	498.931 ± 62.834	483.913 ± 55.271
	Van der Waals	-0.002 ± 0.001	-0.018 ± 0.008	-0.037 ± 0.030
average	electrostatic	-145.761 ± 15.196	472.669 ± 58.079	440.619 ± 74.606
	Van der Waals	-0.005 ± 0.001	-0.007 ± 0.007	-0.018 ± 0.003

Table S6: Secondary structures of the dodecamer. Only the chains in contact with the membrane were taken into account. The last row refers to the secondary structure of the starting structure used in the simulation with the membrane.

Trajectory	Beta (%)	Helix (%)	Turn (%)	Coil (%)
1	6.4	26.6	25.1	41.9
2	0.0	15.7	24.6	59.7
3	4.7	25.2	23.4	46.7
4	3.9	32.6	25.3	38.2
5	5.8	21.8	29.2	43.2
6	7.2	26.1	24.2	42.5
7	6.6	21.1	27.4	44.9
8	4.2	22.4	35.1	38.3
9	3.9	32.9	22.8	40.4
10	3.3	28.5	29.8	38.4
Average	4.6 ± 1.9	25.3 ± 4.8	26.7 ± 3.4	43.4 ± 5.8
Initial structure	2.76	22.02	28.95	46.27

Table S7: Secondary structures of the mature fibril . Only the chains in contact with the membrane were taken into account. The snapshots taken from trajectories 1, 3, 4, 7, and 9 were skipped because the fibril was far from the membrane. The last row refers to the secondary structure of the initial structure used in the simulation with the membrane.

Trajectory	Beta (%)	Helix (%)	Turn (%)	Coil (%)
2	34.9	1.2	18.6	45.3
5	33.3	0.1	20.6	46.0
6	37.1	0.1	20.4	42.4
8	33.0	0.2	22.9	43.9
10	27.5	2.2	25.8	44.5
Average	33.2 ± 3.2	0.8 ± 0.8	21.7 ± 2.5	44.4 ± 1.2
Initial structure	36.71	0.00	21.59	41.70

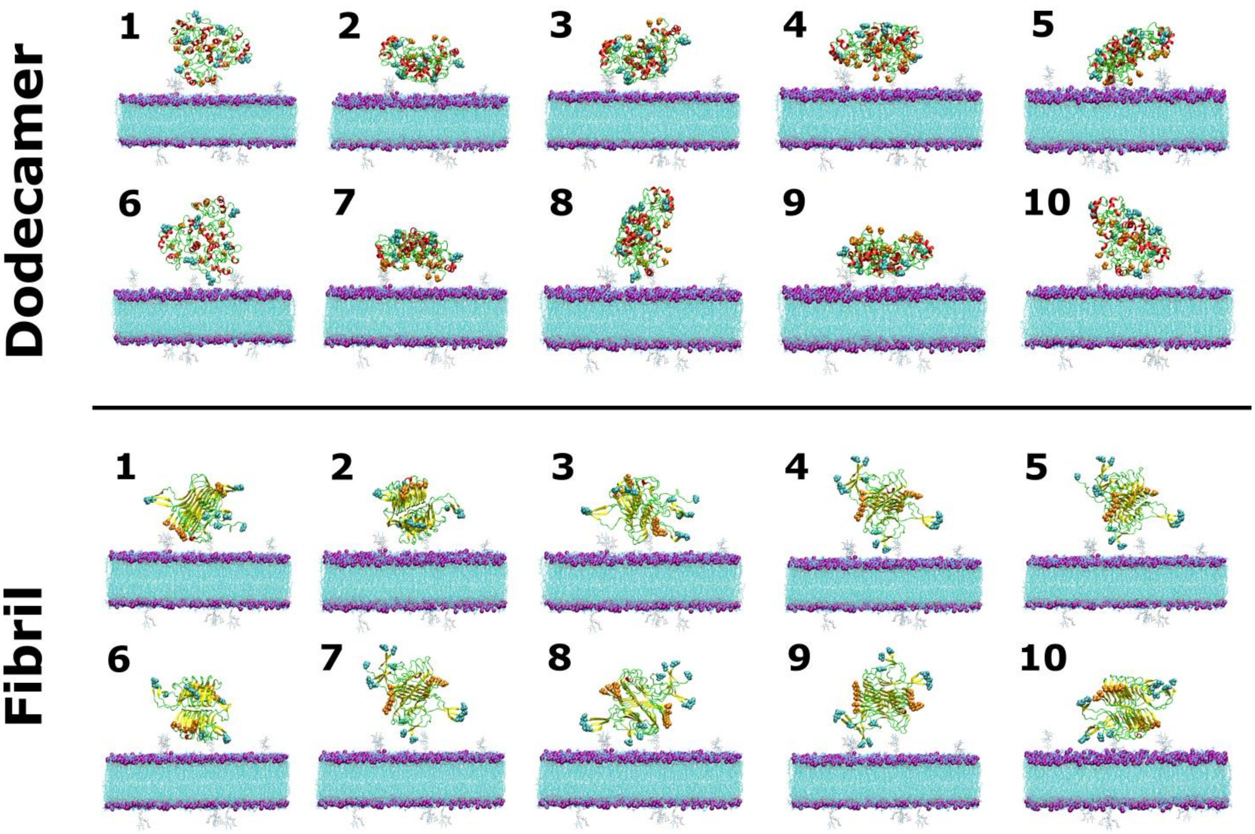

Figure S1. Initial structures used in MD simulations for membrane-dodecamer and membrane-fibril complexes. The structure of the dodecamer and fibril was equilibrated by MD simulation in solution with a duration of 500 ns, as described in the main text.

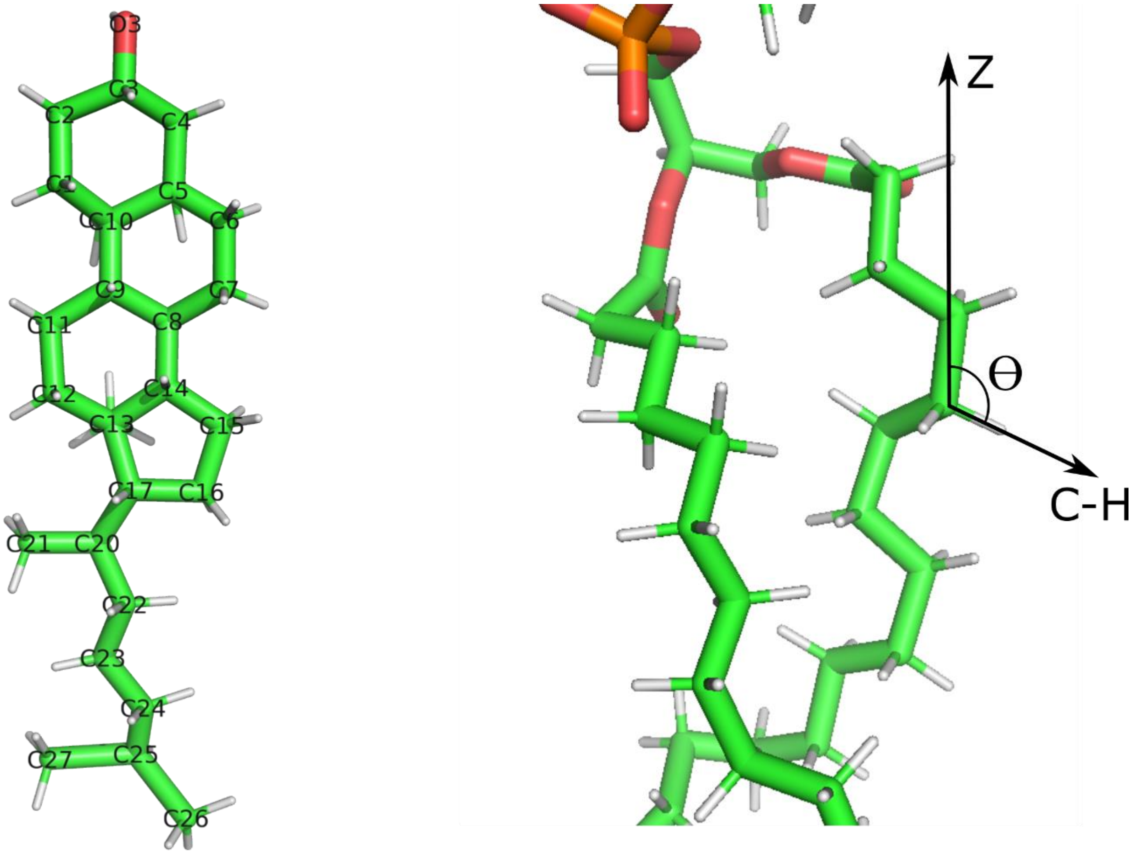

Figure S2: (Left) Atomic numbering for cholesterol. (Right) Definition of the Θ angle between the C-H bond vector and the z-axis.

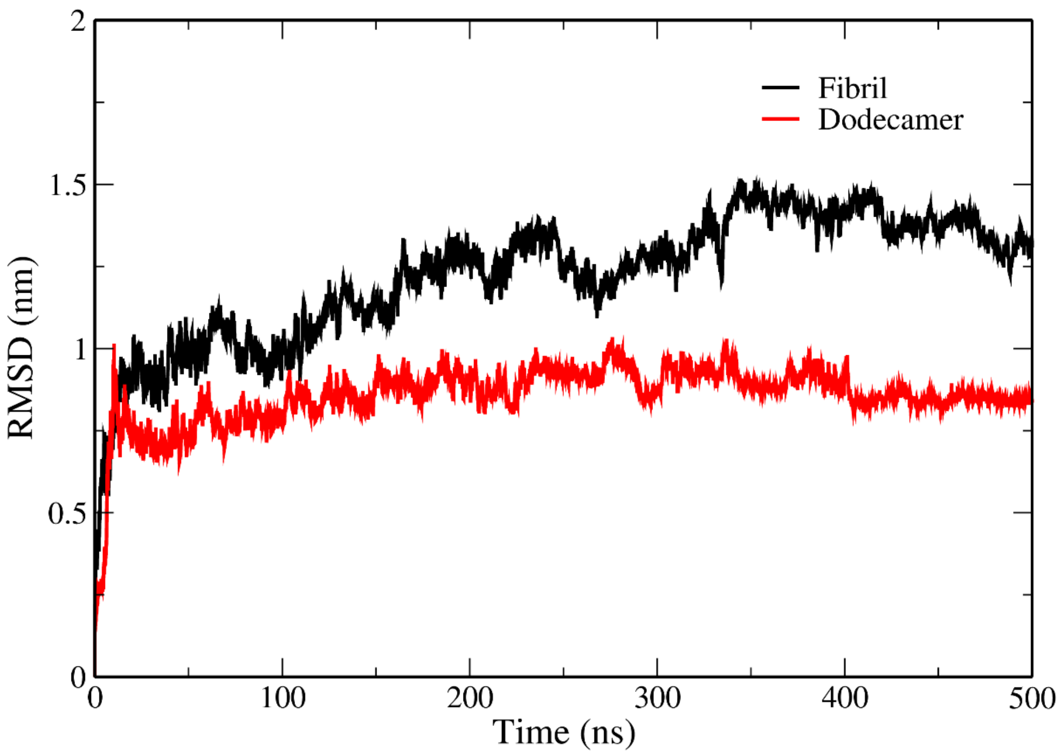

Figure S3: Time dependence of RMSD of C-alpha atoms of dodecamer and mature fibril in solution.

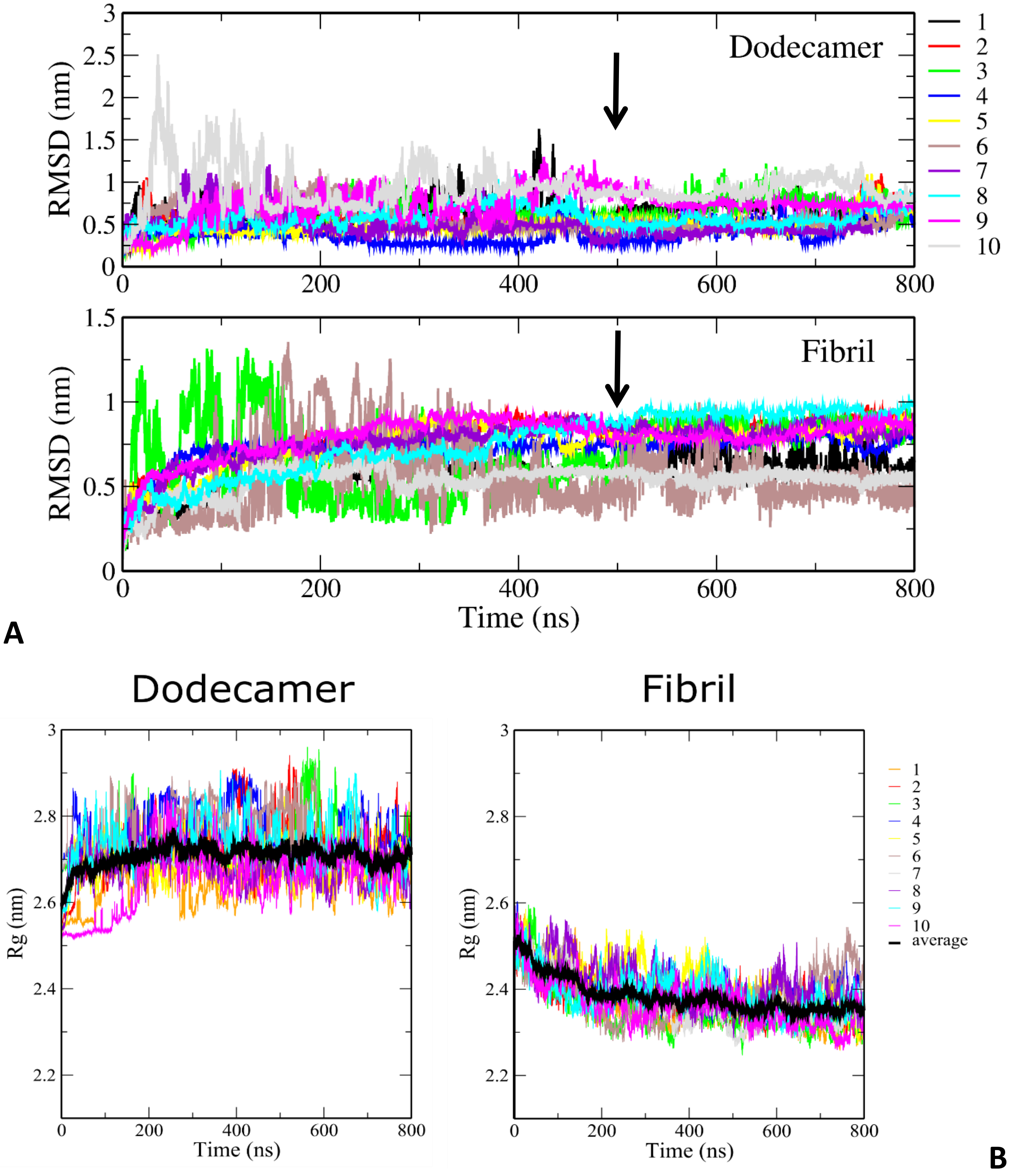
Dodecamer
RMSD (nm)
1
2
3
4
5
6
7
8
9
10
Fibril
RMSD (nm)
Time (ns)
A
Dodecamer
Rg (nm)
Time (ns)
Fibril
Rg (nm)
Time (ns)
1
2
3
4
5
6
7
8
9
10
average
B

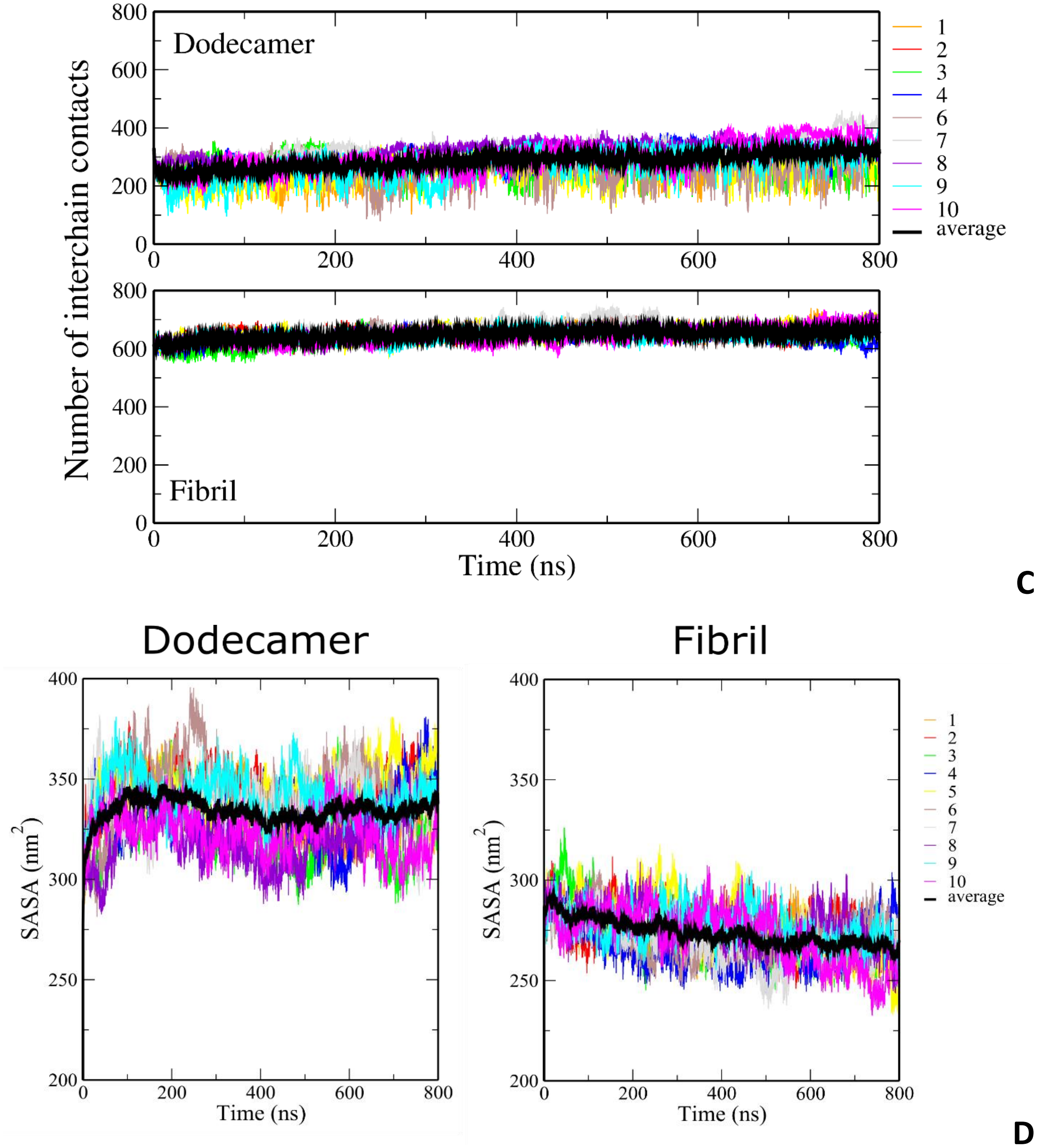

Figure S4: Structural properties of the Aβ dodecamer and fibril: A) Time dependence of the Cα RMSD of Aβ. The results were obtained from 10 MD runs for both complexes. The arrow refers to the equilibration time τ_{eq} = 500 ns, B) As in A but for *R*g, C) As in A but for the number of contacts between Aβ chains (a contact is formed if the distance between centers of mass of two residues is ≤ 6.5 Å), and D) As in A but for SASA.

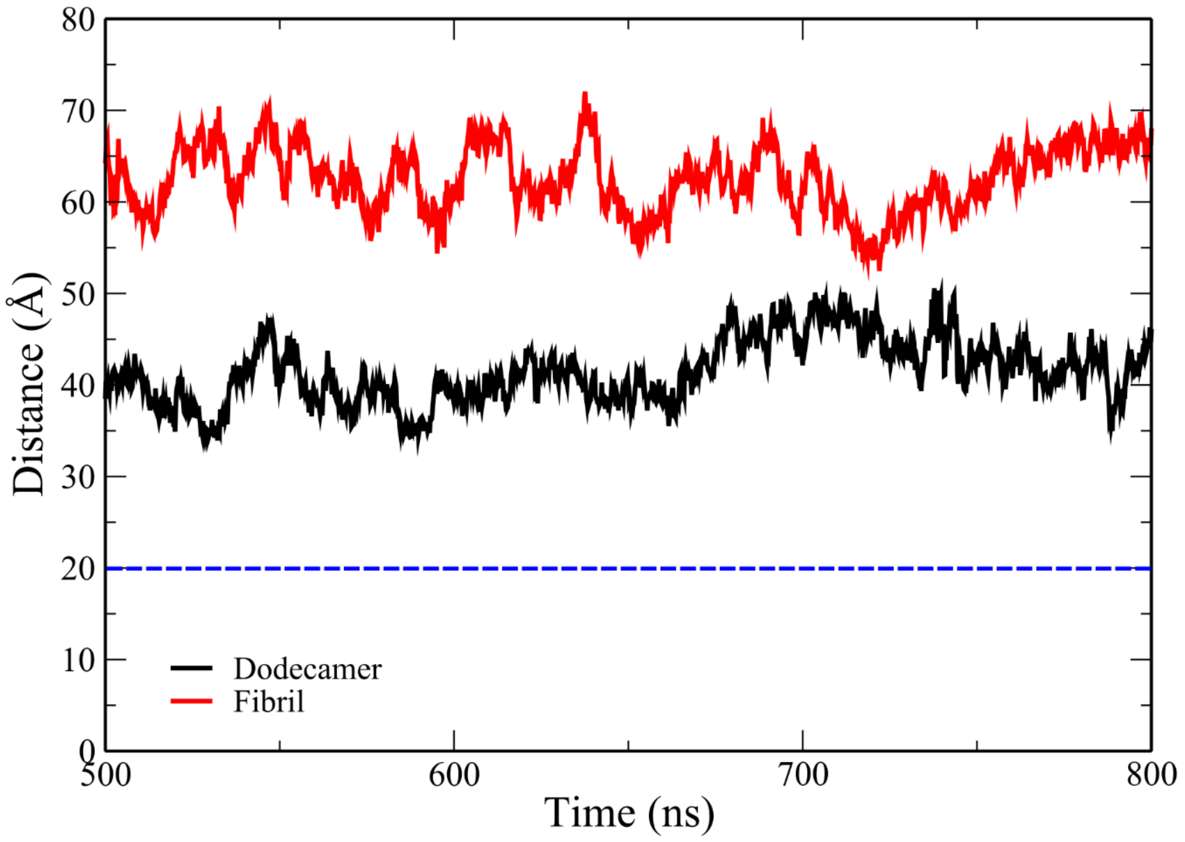

Figure S5: Average minimum distance between the dodecamer (black) and fibril (red) and the membrane center along the z-axis. The dashed line represents the membrane surface. Averaging over all snapshots we obtained the minimum distance of 40.00 ± 3.93 and 62.83 ± 4.91 Å for the dodecamer and fibril, respectively.

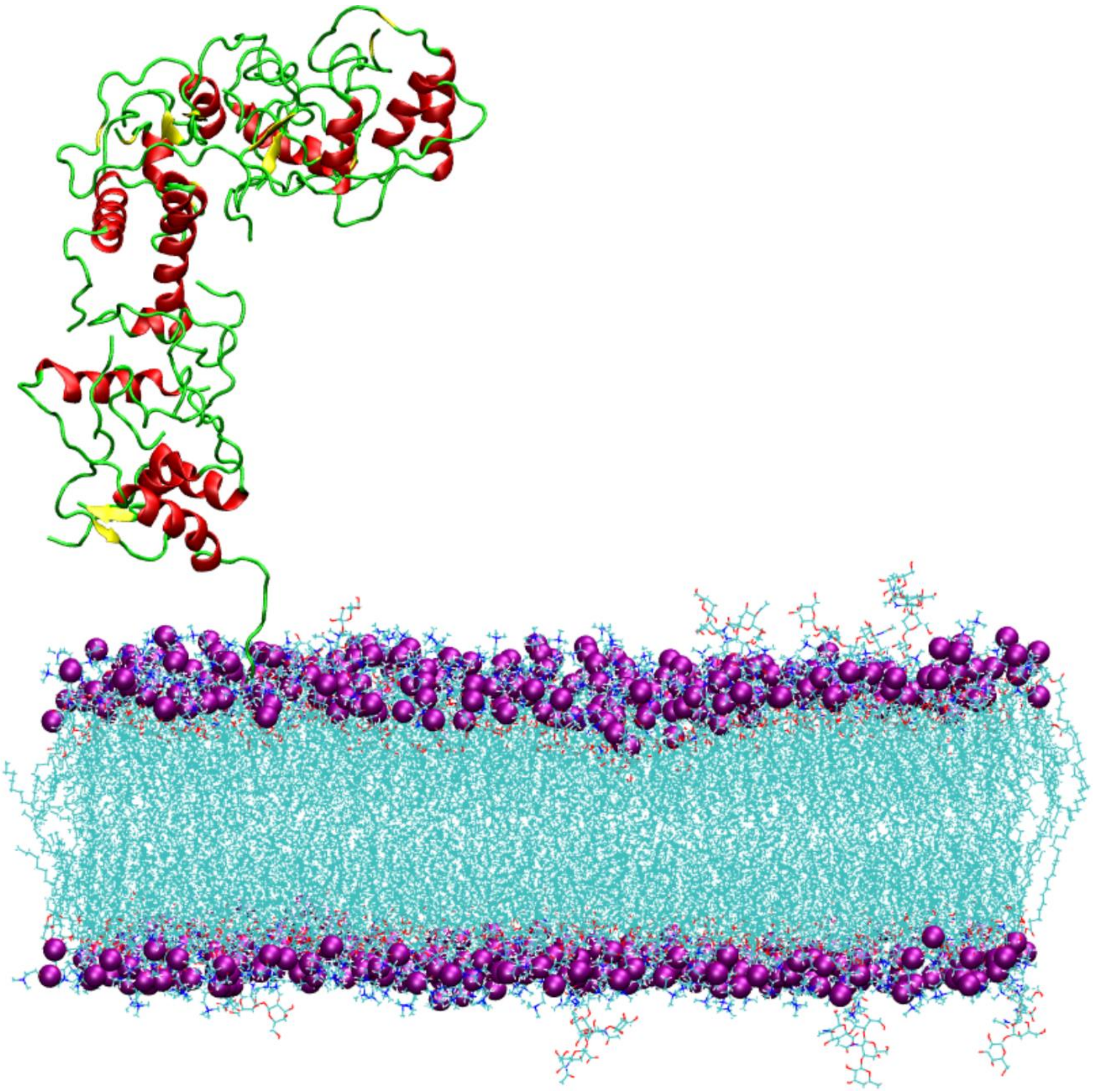

Figure S6: A snapshot showing the touch of the Aβ42 dodecamer to the membrane surface. This snapshot was taken from trajectory 4 at 530 ns.

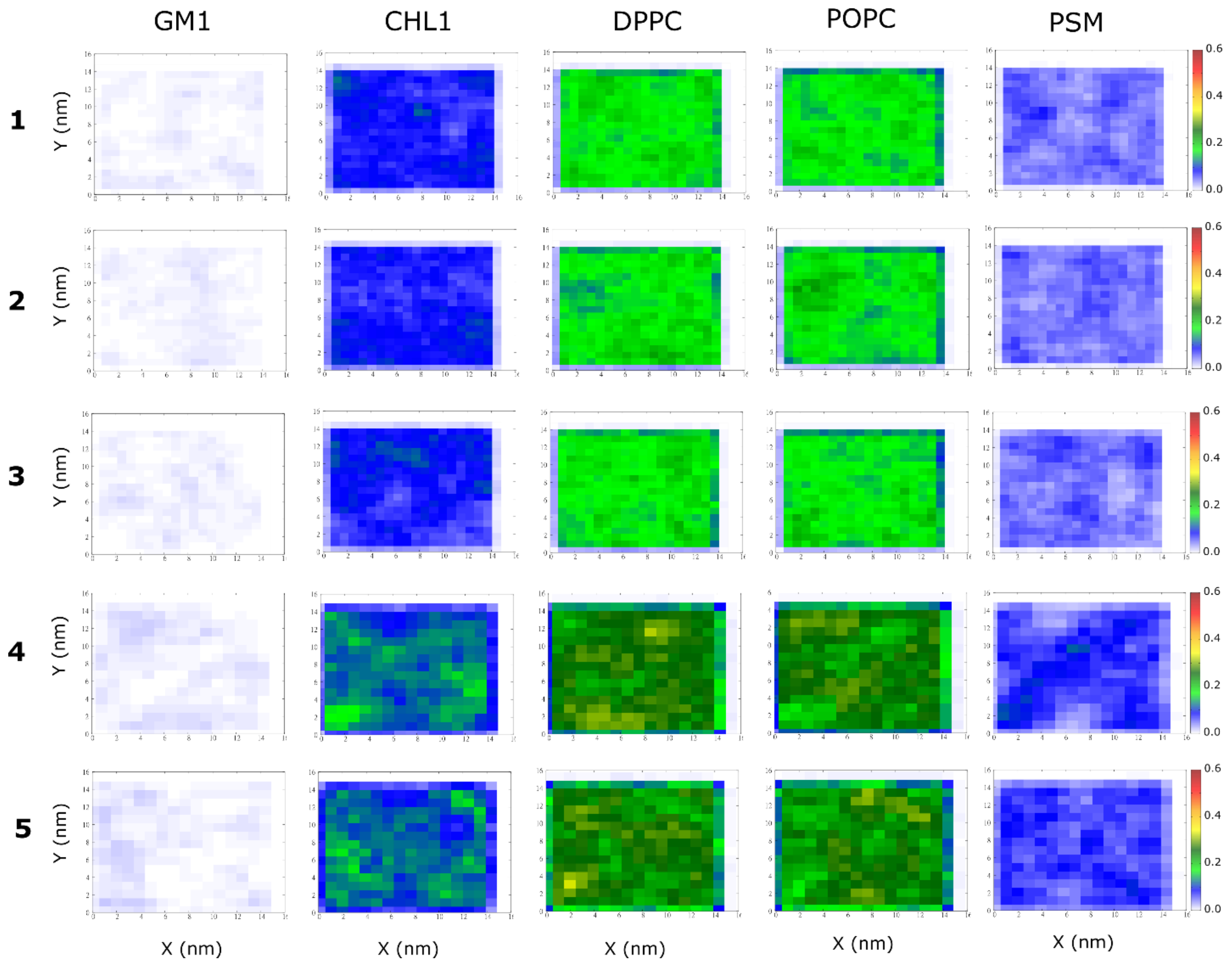

Figure S7: Distribution of lipid molecules in trajectories 1-5 of the membrane-dodecamer complex.

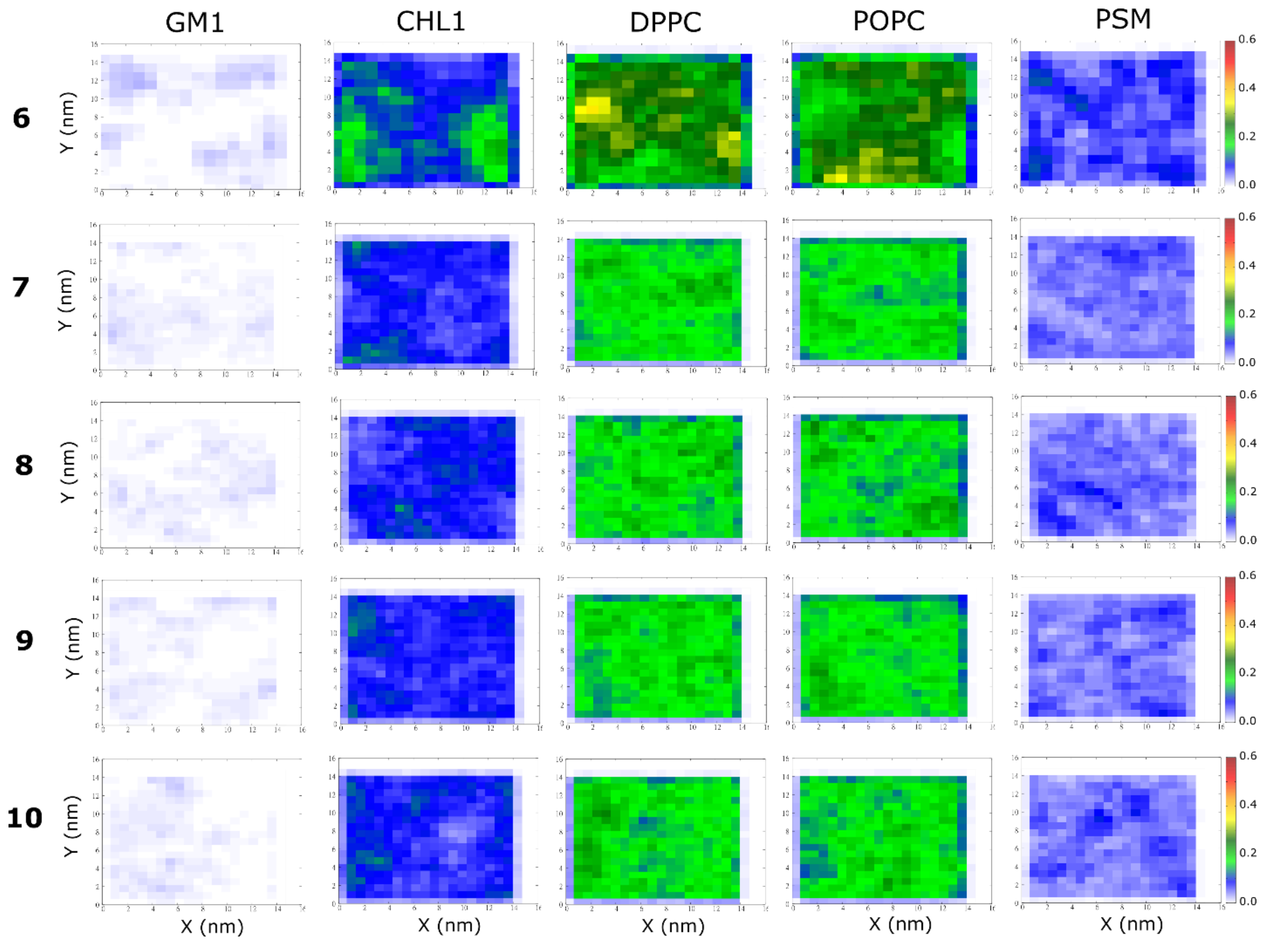

Figure S8: Distribution of lipid molecules in trajectories 6-10 of the membrane-dodecamer complex.

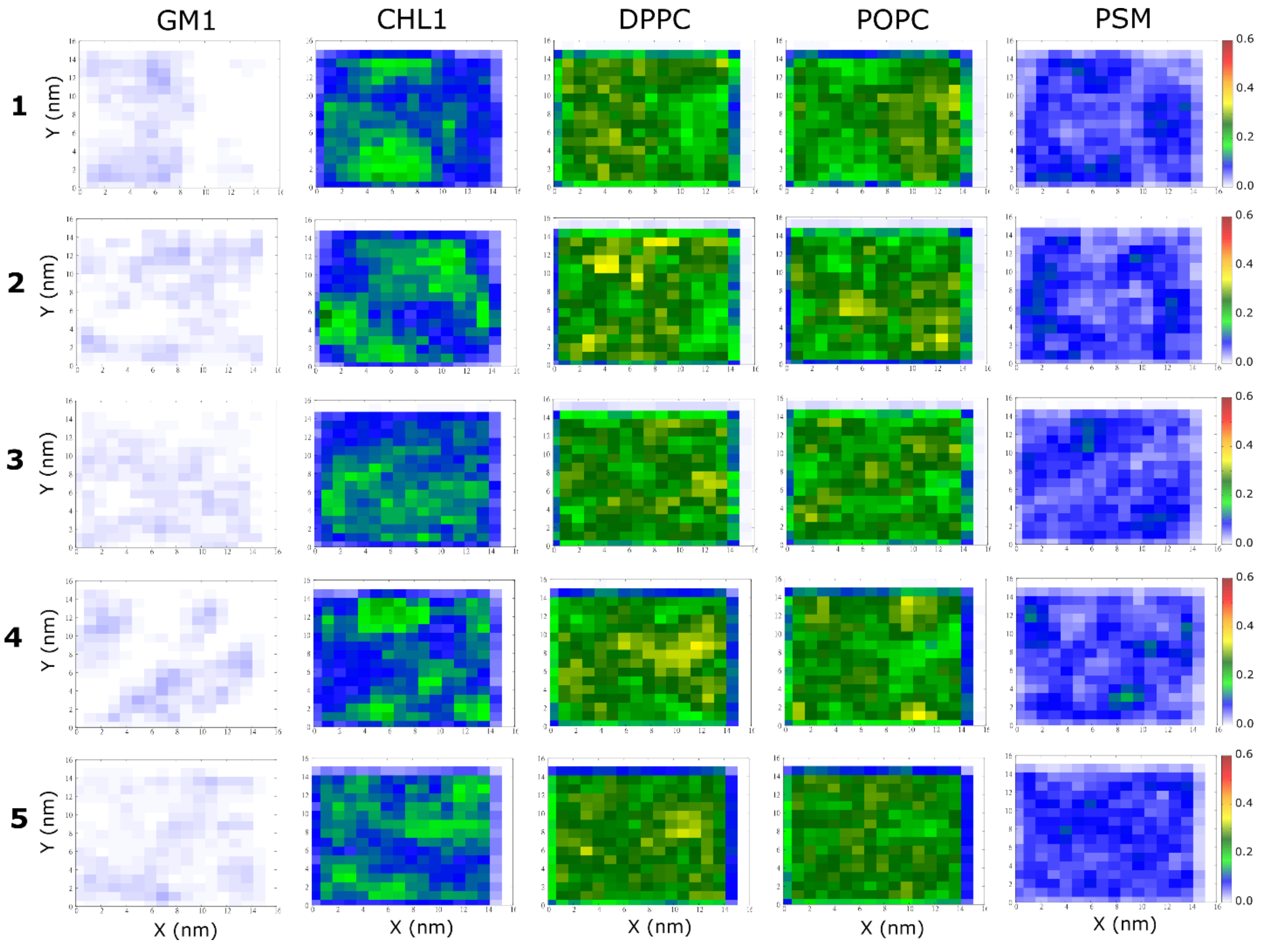

Figure S9: Distribution of lipids in trajectories 1-5 of the membrane-fibril complex.

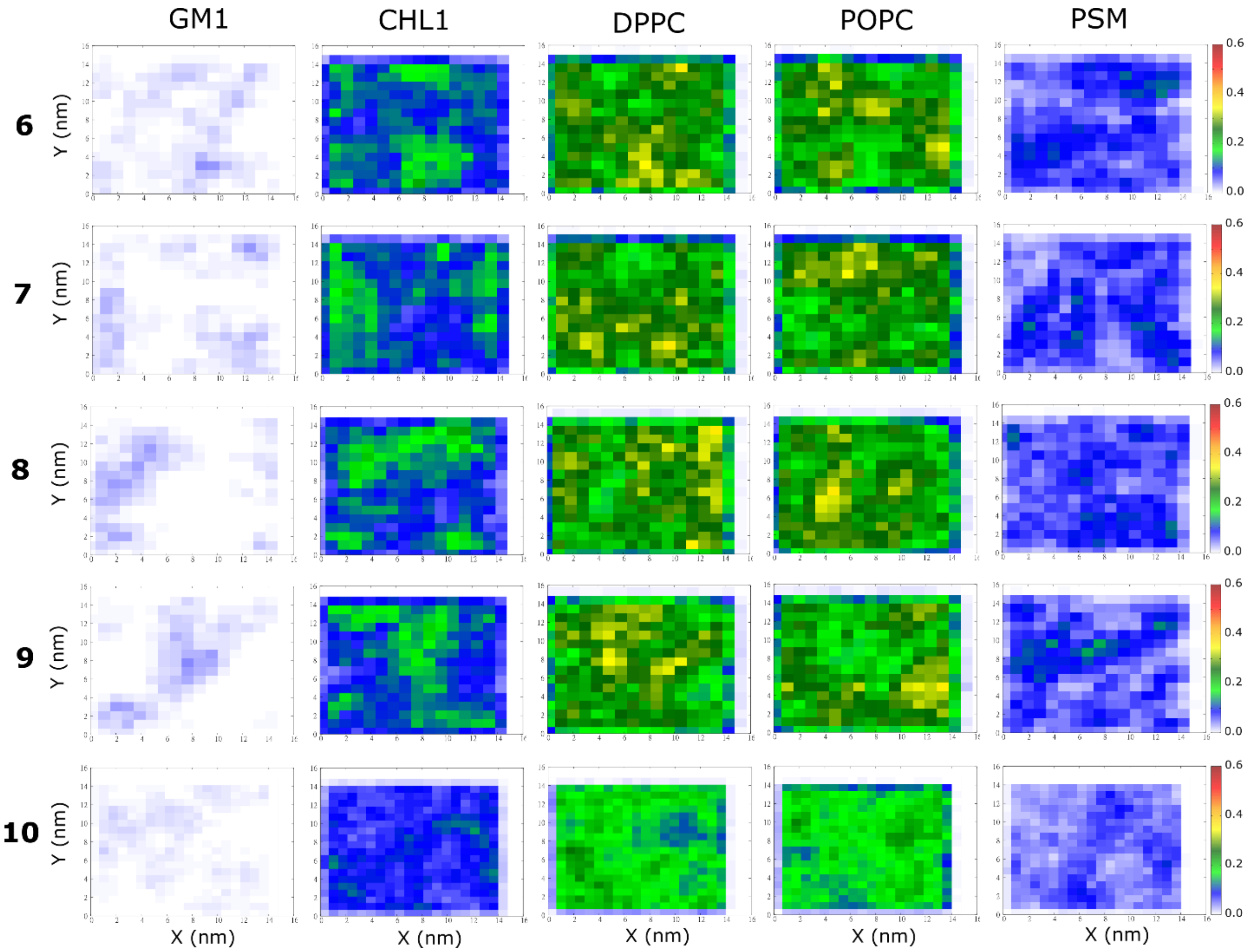

Figure S10: Distribution of lipids in trajectories 6-10 of the membrane-fibril complex.

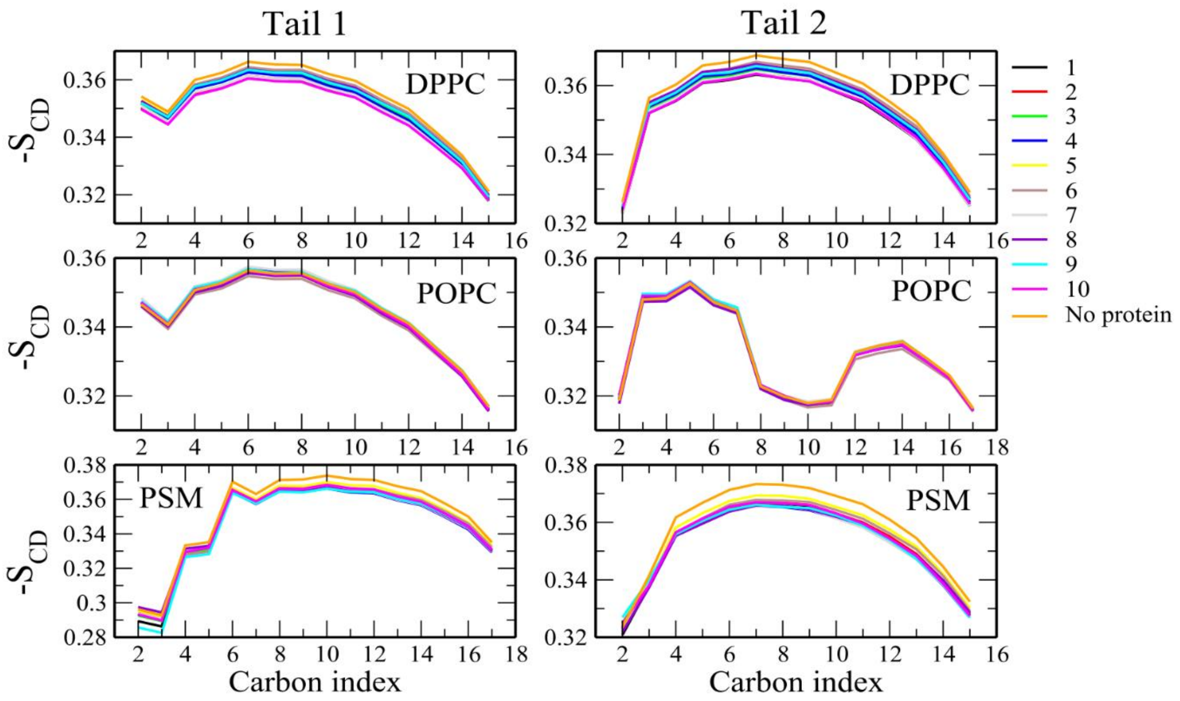

Figure S11: Tail order parameters of lipid molecules in the presence of Aβ42 dodecamer.

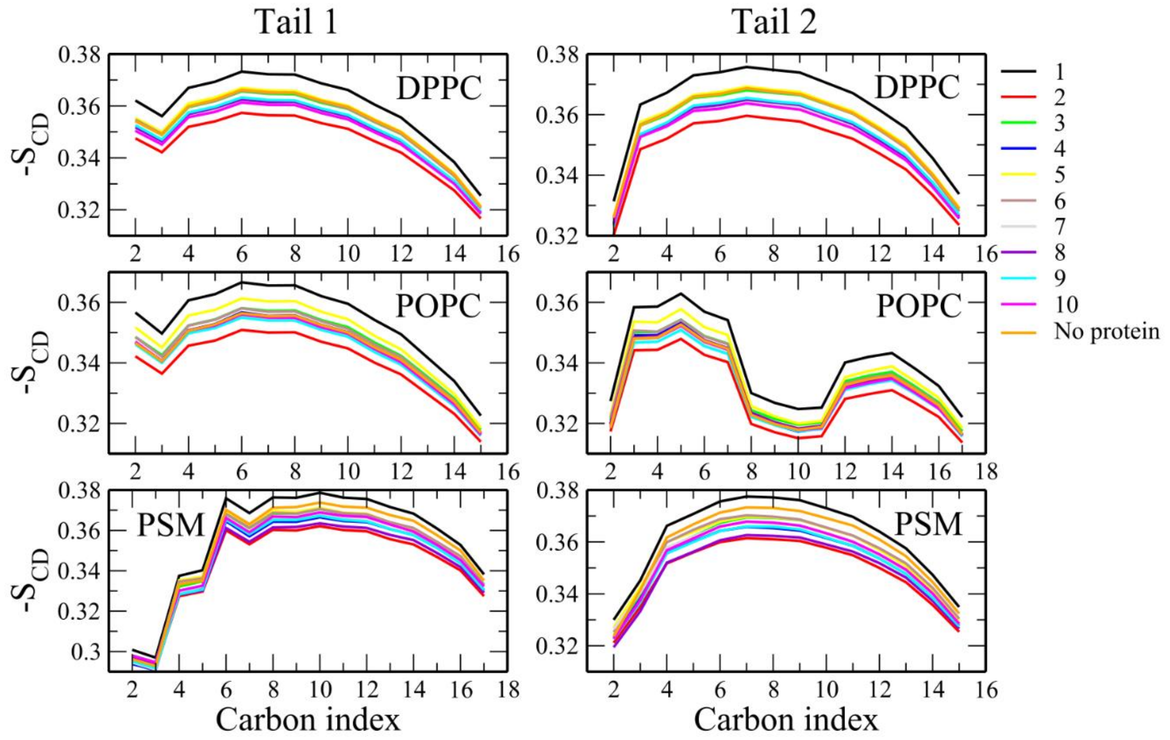

Figure S12: Tail order parameters of lipid molecules in the presence of Aβ42 fibril.

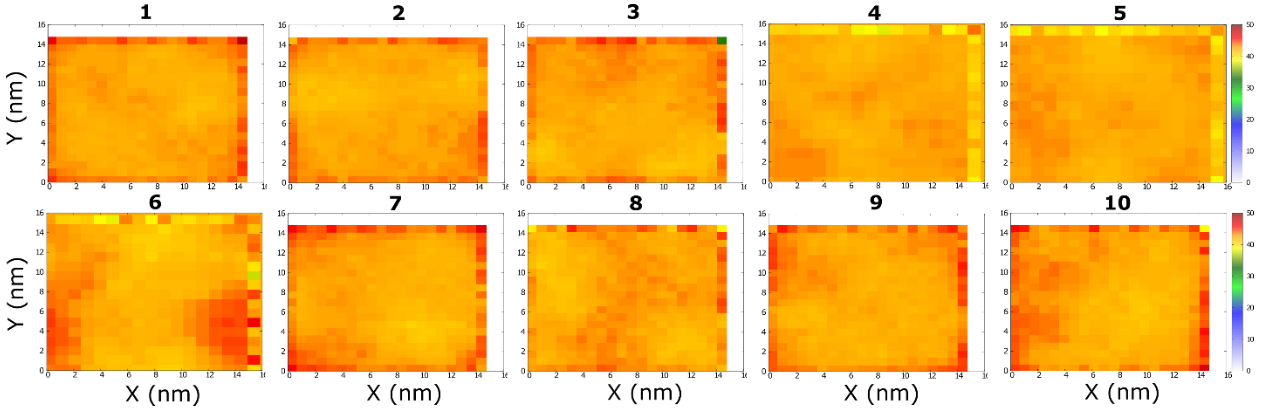

Figure S13: Distribution of the membrane thickness in the presence of Aβ42 dodecamer.

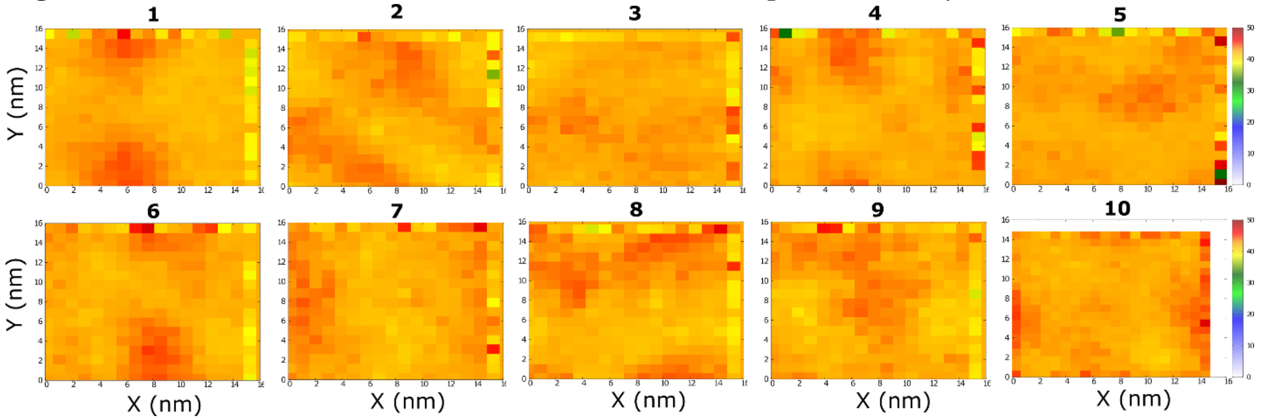

Figure S14: Distribution of the membrane thickness in the presence of Aβ42 fibril.

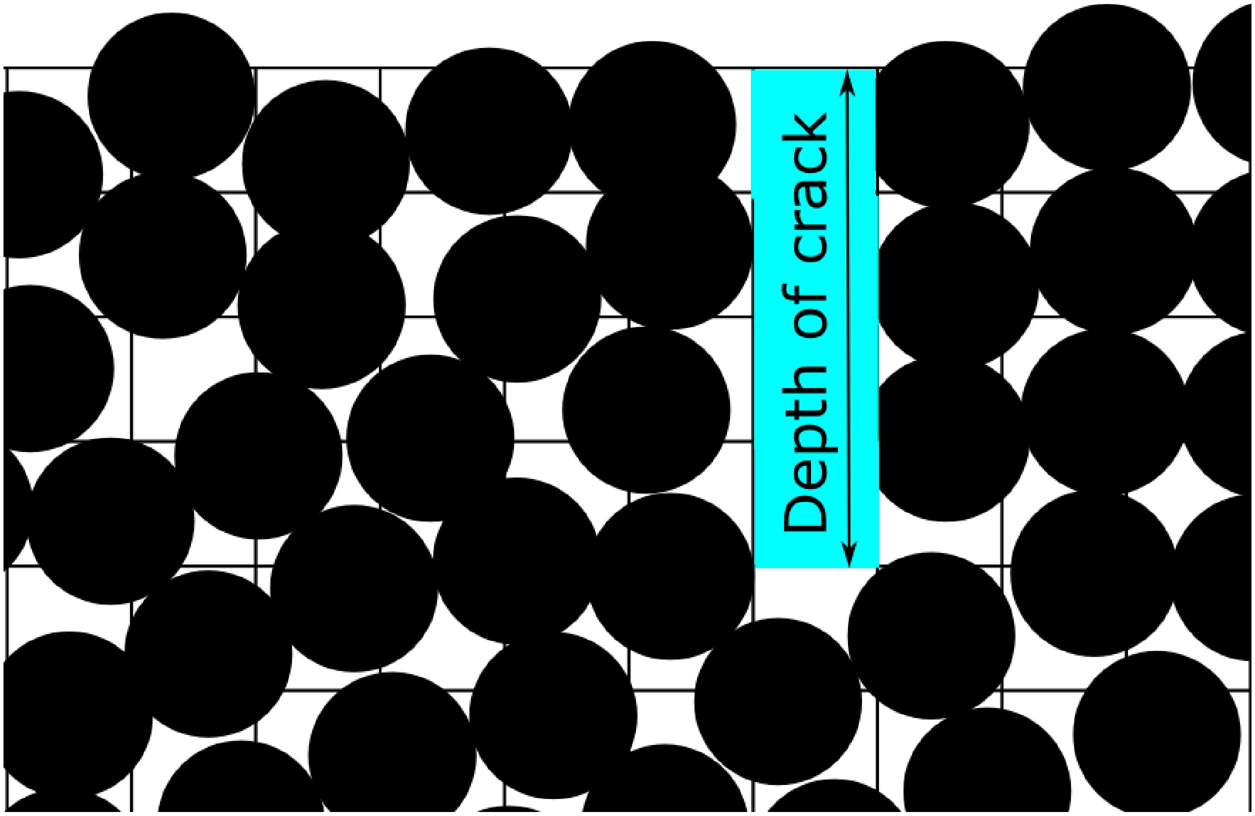

Figure S15. Definition of the crack depth.

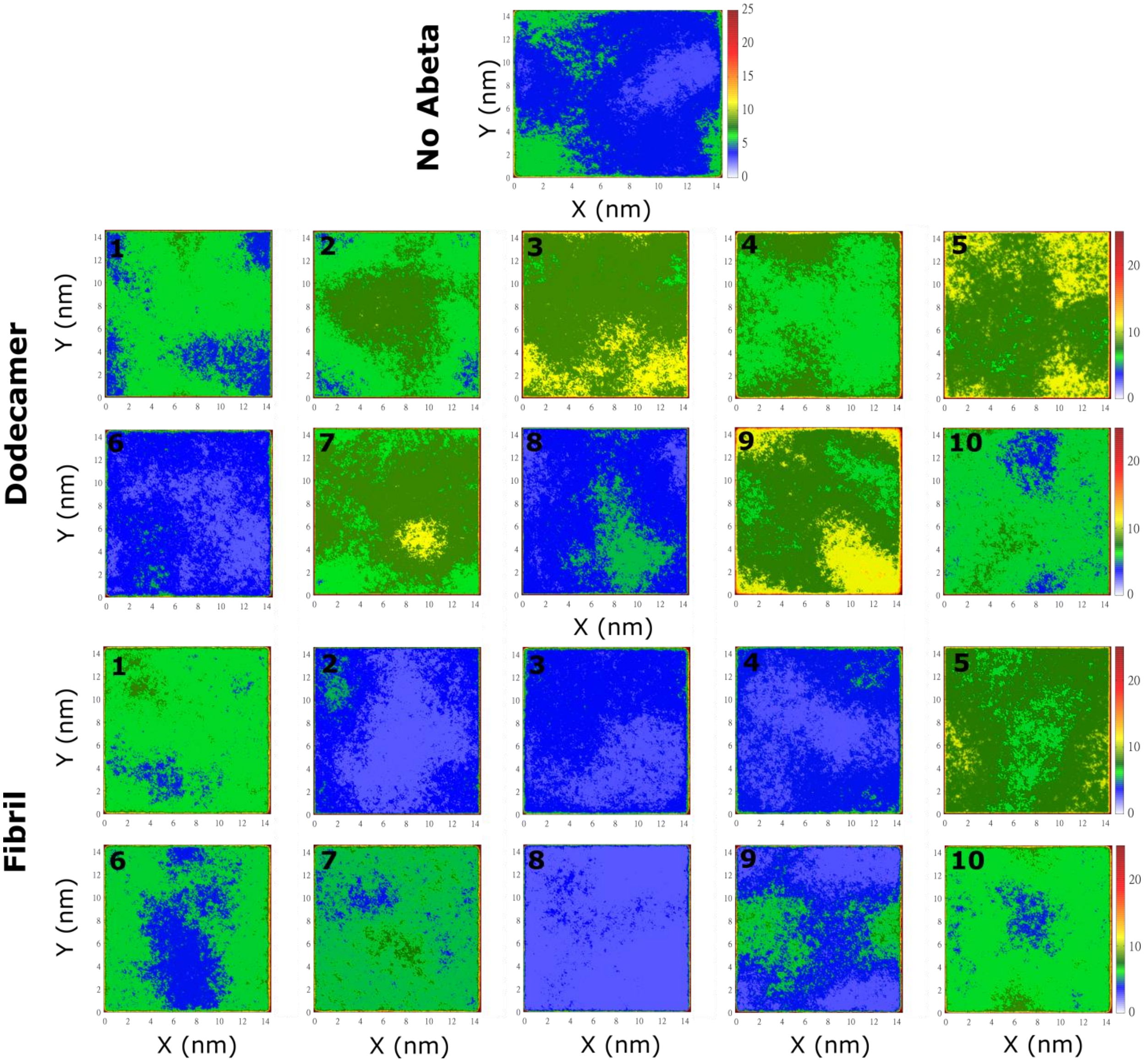

Figure S16: Distribution of crack depths on the membrane surface.
